# The PTK7-Related Transmembrane Proteins Off-track and Off-track 2 Are Co-receptors for *Drosophila* Wnt2 Required for Male Fertility

**DOI:** 10.1371/journal.pgen.1004443

**Published:** 2014-07-10

**Authors:** Karen Linnemannstöns, Caroline Ripp, Mona Honemann-Capito, Katja Brechtel-Curth, Marie Hedderich, Andreas Wodarz

**Affiliations:** 1 Stem Cell Biology, Institute for Anatomy and Cell Biology, University of Goettingen, Goettingen, Germany; 2 Institute for Developmental Biochemistry, University of Goettingen, Goettingen, Germany; Harvard Medical School, Howard Hughes Medical Institute, United States of America

## Abstract

Wnt proteins regulate many developmental processes and are required for tissue homeostasis in adult animals. The cellular responses to Wnts are manifold and are determined by the respective Wnt ligand and its specific receptor complex in the plasma membrane. Wnt receptor complexes contain a member of the Frizzled family of serpentine receptors and a co-receptor, which commonly is a single-pass transmembrane protein. Vertebrate protein tyrosine kinase 7 (PTK7) was identified as a Wnt co-receptor required for control of planar cell polarity (PCP) in frogs and mice. We found that flies homozygous for a complete knock-out of the *Drosophila* PTK7 homolog *off track* (*otk*) are viable and fertile and do not show PCP phenotypes. We discovered an *otk* paralog (*otk2*, *CG8964*), which is co-expressed with *otk* throughout embryonic and larval development. Otk and Otk2 bind to each other and form complexes with Frizzled, Frizzled2 and Wnt2, pointing to a function as Wnt co-receptors. Flies lacking both *otk* and *otk2* are viable but male sterile due to defective morphogenesis of the ejaculatory duct. Overexpression of Otk causes female sterility due to malformation of the oviduct, indicating that Otk and Otk2 are specifically involved in the sexually dimorphic development of the genital tract.

## Author Summary

Wnts are secreted, growth factor-like proteins that are important for the development of many tissues and organs in animals. They are also required in adult animals and humans for controlling the balance between growth and differentiation. Wnts are bound at the cell surface by Wnt receptors, which are dimers composed of a Frizzled protein and a co-receptor. Here we have analyzed the *Drosophila* Wnt co-receptors Off-track (Otk) and Off-track 2 (Otk2), which are closely related to vertebrate Protein tyrosine kinase 7 (PTK7). We found that in contrast to PTK7 in mice and frogs, which controls planar cell polarity (PCP), Otk and Otk2 together are needed in males for development of the ejaculatory duct, a tube-like organ that transports the mature sperm. Our data furthermore indicate that Otk and Otk2 are co-receptors for Wnt2. The sterile phenotype of *Wnt2* mutant males is not identical to that of *otk, otk2* double mutants, so additional Wnts may be involved in this process. Interestingly, the function of Wnt2 in male fertility appears to be evolutionarily conserved, because male mice mutant for Wnt7A, the vertebrate homolog of *Drosophila* Wnt2, are sterile due to abnormal development of the vas deferens, which corresponds to the fly ejaculatory duct.

## Introduction

Wnt proteins bind at the cell surface to transmembrane receptors, which transduce the signal to downstream components of the various branches of Wnt signal transduction [1]. In addition to members of the Frizzled receptor family, which were the first Wnt receptors to be identified [2], Wnts were also shown to bind to the transmembrane proteins low density lipoprotein receptor-related protein 5/6 (LRP5/6) [3], [4], receptor tyrosine kinase-like orphan receptors 1/2 (Ror1/2) [5], [6], related to receptor tyrosine kinase (Ryk) [7]–[9], muscle specific kinase (MuSK) [10], syndecan [11] and protein tyrosine kinase 7 (PTK7) [12], reviewed in [13]. Some of these Wnt co-receptors form a receptor complex together with a Frizzled protein, whereas others are capable of binding Wnts in the absence of Frizzleds. In general, it is thought that the presence of different classes of Wnt receptors and co-receptors on the cell surface increases the specificity of the interaction of Wnts with their target cells and also serves to channel the Wnt signal into either the canonical, β-catenin-dependent branch or one of the so-called non-canonical branches of Wnt signaling [14], [15].

One Wnt co-receptor of particular interest is PTK7. PTK7 is required for the control of planar cell polarity (PCP) in vertebrates. Mice mutant for PTK7 show an open neural tube, defects in convergent extension movements during gastrulation and polarity defects of inner ear hair cells, which are classical PCP phenotypes in vertebrates [16]–[21]. PCP phenotypes were also observed upon knock-down or mutation of PTK7 in *Xenopus* [18] and zebrafish [22]. PTK7 knock-down in *Xenopus* furthermore caused defects in migration of cranial neural crest cells [23], very similar to animals in which the function of Dishevelled, an intracellular component of Wnt signaling, has been impaired [24].

Regarding the mechanism of how PTK7 controls PCP, it was clearly demonstrated in *Xenopus* that PTK7 interacts physically with several components of Wnt signal transduction. As expected for a putative Wnt co-receptor, PTK7 binds to Wnt proteins and forms a complex with Frizzled7, LRP6 and Dishevelled [12], [23], [25]. Somewhat unexpectedly, although PTK7 is supposed to promote the non-canonical PCP branch of Wnt signaling, it was found to bind to the canonical ligands Wnt3A and Wnt8 but failed to bind the non-canonical ligands Wnt5A and Wnt11 [12]. Additional experiments showed that co-overexpression of PTK7 with either Wnt3A or with Wnt8 blocked the capability of these Wnts to induce a second body axis in *Xenopus*, consistent with a function for PTK7 in suppression of canonical Wnt signaling, which may be essential for activation of non-canonical signaling [12]. This interpretation was recently strengthened by similar findings in zebrafish [22], but is in conflict with other studies coming to essentially the opposite conclusion, according to which PTK7 promotes canonical Wnt signaling [25], [26].

In *Drosophila*, mutations in genes that were reported to interact physically and functionally with PTK7 in vertebrates, including *frizzled* (*fz*) [27], [28], *dishevelled* (*dsh*) [29] and *Van Gogh/strabismus* (*Vang*) [30] all show PCP phenotypes. Surprisingly, until recently this was not reported for the proposed PTK7 homolog in *Drosophila*, encoded by the gene *off-track* (*otk*) [31], [32]. These studies reported that a loss-of-function mutation of *otk* is embryonic lethal and causes axon pathfinding defects of certain embryonic motor nerves [32] and targeting defects of outer photoreceptor axons to the lamina in the developing eye [31]. Only recently it was reported that *otk* mutant embryos show cuticular defects pointing to a function in the determination of segment polarity [12]. These authors furthermore showed that Otk can bind Wnt4 and that mutation of *otk* blocks the occurrence of cuticular patterning defects observed upon overexpression of Wnt4 [12]. Together, these data led to the conclusion that Otk is a receptor for Wnt4 required for its function in embryonic patterning.

Due to these obvious inconsistencies in the published literature we have reinvestigated the function of PTK7/Otk in *Drosophila*. We found that there are in fact two homologs of PTK7 in the fly that were most likely the result of a gene duplication that occurred only in the genus *Drosophila* but not in other insect species. In addition to the previously described *otk* gene we identified *otk2* to encode a second close homolog of PTK7. *otk2* is positioned directly adjacent to *otk* on the second chromosome and is expressed in a pattern identical to *otk*. We generated null mutations for *otk, otk2* and a double mutation that deletes both genes together. In contrast to the published literature, we found that both single mutants were homozygous viable and fertile without showing any of the previously reported mutant phenotypes. The *otk, otk2* double mutant was homozygous viable but male sterile due to defective morphogenesis of the ejaculatory duct. The male sterile phenotype caused us to investigate potential interactions of both Otk and Otk2 with Wnt2, which is also required for male fertility [33]. We indeed show that embryonic expression of *otk* depends on Wnt2 and that both Otk and Otk2 coimmunoprecipitate with Wnt2, Fz and Fz2, indicating that Otk and Otk2 are co-receptors for Wnt2. Our results provide important information for unraveling the system of Wnt ligands and their specific receptors involved in male fertility. Furthermore, they may have implications for studying male fertility in humans, since mutation of Wnt7a, the mouse homolog of *Drosophila* Wnt2, also causes male and female sterility [34].

## Results

### *off-track* and the adjacent gene *off-track2* (*otk2, CG8964*) are paralogs evolved by gene duplication

Previous analyses of *otk*, the proposed *Drosophila* homolog of PTK7, did not reveal any function in the control of PCP or β-catenin-dependent Wnt signaling [31], [32]. Given that mutation of PTK7 causes strong loss-of-function phenotypes in vertebrates [18]–[20], [23], we speculated that there may be a second gene in the fly genome that could function redundantly with *otk*. Indeed, when we performed a BLAST search with the protein sequence of Otk we found that the protein encoded by the gene *CG8964* is closely related to Otk (53% identity over 427 amino acids, Figure S1). *CG8964* is located right next to *otk* (see http://flybase.org/) on the second chromosome [2R: 7,910,651–7,912,775(-)], suggesting that it is the result of a gene duplication. Therefore, we named the gene *CG8964 off-track2* (*otk2*).

**Figure 1:**
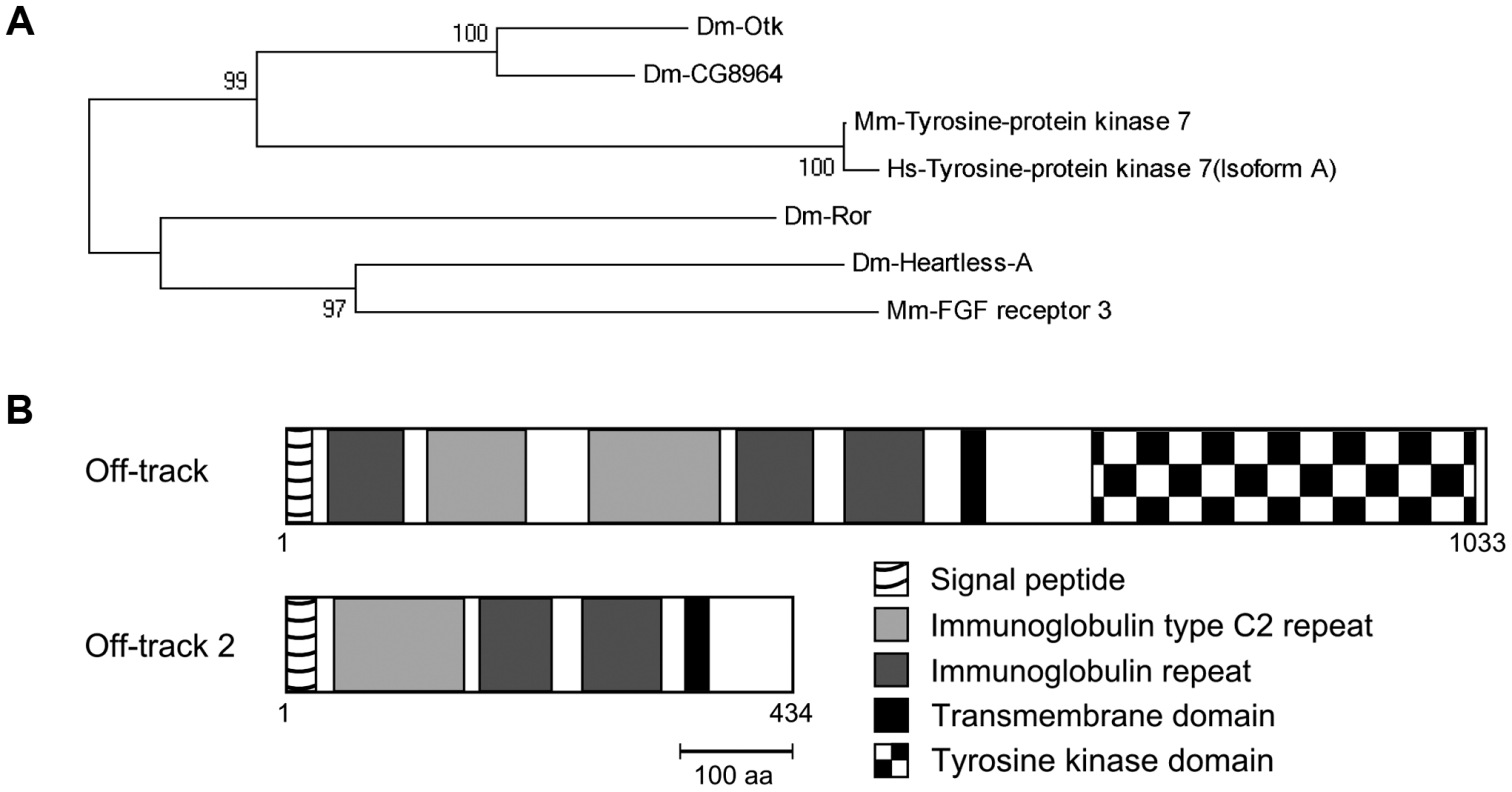
Off-track (Otk) and Off-track2 (CG8964, Otk2) are paralogs evolved by gene duplication. (A) Phylogenetic tree representation of an alignment of sequences from different species homologous to *Drosophila* Otk. Blast-P of Dm-Otk in *Drosophila* returned Dm-CG8964 (Otk2) as the best hit; Dm-Ror and Dm-Heartless-A were the second and third hit, repectively. Blast-P of Dm-Otk in Mouse returned Mm-PTK7 as the best hit, while Mm-FGF receptor3 was the second best hit. Blast-P of Dm-Otk in Human returned Hs-PTK7 as the best hit. ClustalW alignment of these sequences and Neighbor-Joining tree confirms that the PTK7 branch is separated from the other proteins by a bootstrap value of 100. In this branch, two *Drosophila* genes, but only one in the other species can be found. (B) Protein structures of Otk and Otk2. Domains were predicted using the SMART sequence analysis tool. Dm, *Drosophila melanogaster*; Mm, *Mus musculus*; Hs, *Homo sapiens*.

Phylogenetic analysis confirmed that *Drosophila otk* and *otk2* are indeed two paralogs of the single *PTK7* gene in mouse and human (Figure 1A). To test whether the gene duplication is specific for *Drosophila* or occurred also in other arthropods, the sequences from different arthropod species homologous to *Drosophila* Otk and Otk2 were analyzed. The resulting phylogenetic tree clearly shows that two Otk paralogs can only be found in *Drosophila* species, but not in other arthropod species (Figure S2). Hence, a lineage specific duplication has generated two PTK7 homologs in *Drosophila*.

Otk is a single-pass transmembrane protein of 114 kD consisting of five extracellular immunoglobulin-like domains, a single transmembrane domain and a kinase homology domain (Figure 1B). Its paralog Otk2 has a molecular weight of 48 kD and only comprises three immunoglobulin-like domains, a single transmembrane domain and a short cytoplasmic domain of 69 amino acids (Figure 1B). To answer the question which parts of the much shorter Otk2 sequence correspond to which parts of the Otk sequence, a dot plot was performed, visualizing matching residues in both sequences. This analysis demonstrated that nearly the entire Otk2 sequence (except for the signal peptide) matches to a contiguous stretch within the Otk sequence (Figure S1B), ranging from the third immunoglobulin-like domain to the beginning of the kinase homology domain.

### Off-track and Off-track2 form homo-and heterooligomers and bind to Frizzled

**Figure 2:**
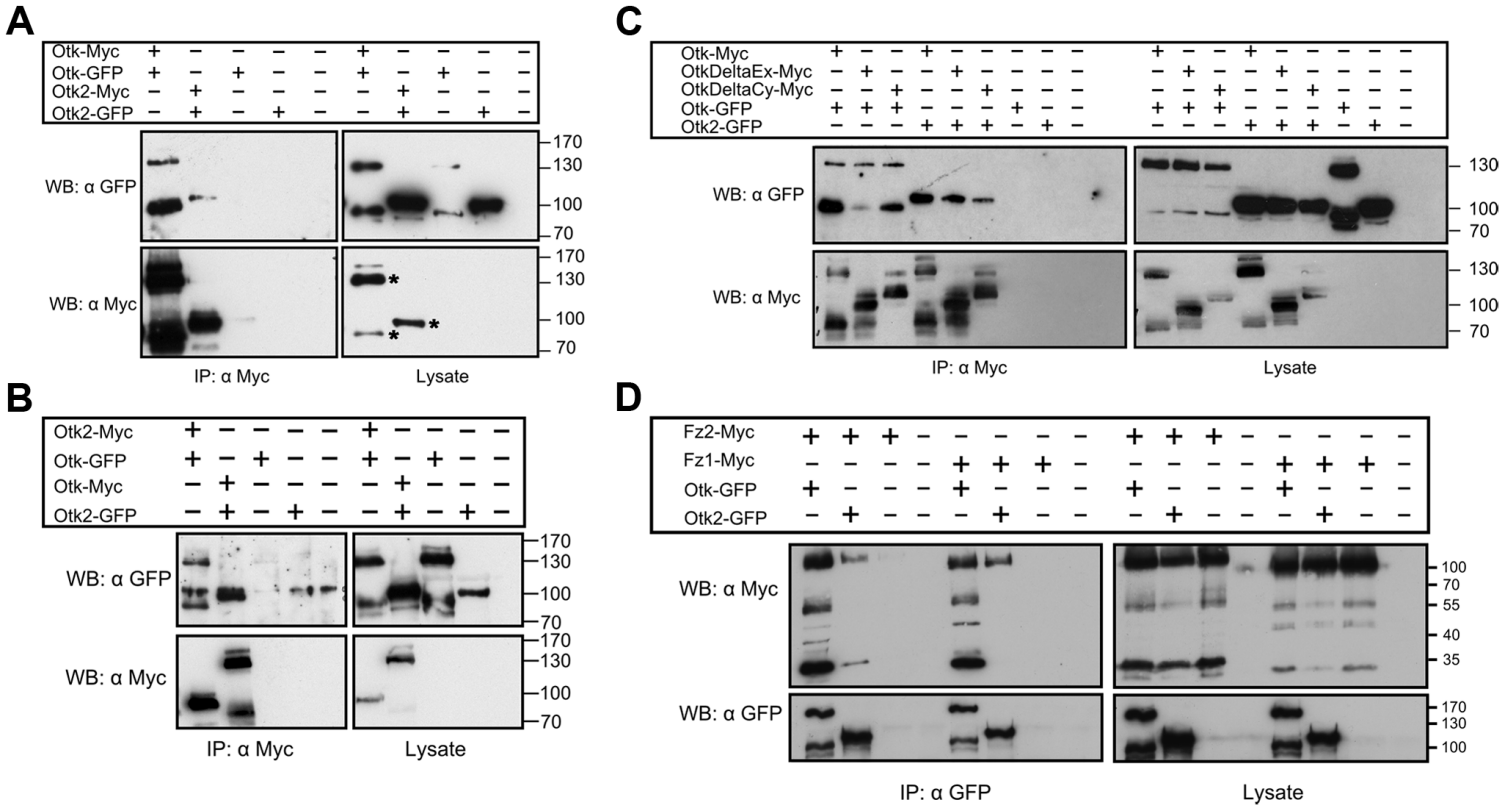
Biochemical interactions of Off-track and Off-track2. (A, B) Otk and Otk2 form homooligomers and heterooligomers. Otk-Myc or Otk2-Myc and Otk-GFP or Otk2-GFP expression vectors were transfected as indicated in *Drosophila* S2r+ cells. Relevant bands corresponding to tagged Otk and Otk2 are marked by an asterisk in the bottom right panel of (A). (C) Homo- and heterodimerization of Otk and Otk2 requires the transmembrane domain. Otk-Myc or Myc-tagged Otk deletion contructs and Otk-GFP or Otk2-GFP expression vectors were transfected as indicated in *Drosophila* S2r+ cells. OtkDeltaCy lacks the cytoplasmic domain (aa 776–1033) and OtkDeltaEx lacks the extracellular domain (aa 2–474). (D) Off-track and Off-track2 interact with Frizzled1 and Frizzled2. Otk-GFP or Otk2-GFP and Fz1-Myc or Fz2-Myc expression vectors were transfected as indicated in *Drosophila* S2r+ cells. Cell lysates were immunoprecipitated and analyzed by Western Blot with the indicated antibodies. IP, Immunoprecipitation; WB, Western Blot.

To test whether Otk and Otk2 have the ability to interact in a homo- or heterophilic manner, we performed co-immunoprecipitation experiments with epitope-tagged proteins transiently transfected into S2r+ cells. To test for homooligomerization, Myc- and GFP-tagged Otk constructs were co-overexpressed in S2r+ cells under control of an actin promoter. Cell lysates were immunoprecipitated using anti-Myc antibody. Immunoblotting with anti-GFP antibody demonstrated that Otk-GFP co-precipitated with Otk-Myc (Figure 2A). Interestingly, both Otk-Myc and Otk-GFP consistently migrated as two bands differing by about 50 kD in size in SDS-PAGE (Figure 2), pointing to modification of the Otk protein by posttranslational processing, most likely proteolysis. Co-IP experiments were also performed with Myc- and GFP-tagged Otk2 constructs and showed that Otk2-GFP co-immunoprecipitated with Otk2-Myc as well (Figure 2A). Cells transfected with GFP-tagged constructs together with an empty vector were used as negative controls (Figure 2A). Hence, both Otk and Otk2 are able to form homooligomers.

The existence of the Otk paralog Otk2 in *Drosophila* raised the question of whether the two proteins can also interact with each other. To test for heterooligomerization, Myc-tagged Otk2 and GFP-tagged Otk constructs were co-overexpressed in S2r+ cells. Cell lysates were subjected to anti-Myc IP. Immunoblotting with anti-GFP antibody demonstrated that Otk-GFP co-precipitated with Otk2-Myc (Figure 2B). The reciprocal experiment was performed with Myc-tagged Otk and GFP-tagged Otk2 constructs and showed that Otk2-GFP co-immunoprecipitated with Otk-Myc as well (Figure 2B). From these results we conclude that Otk and Otk2 form both homo- and heterooligomers. Our co-IP experiments do not allow us to state whether these oligomers are dimers or higher order multimers.

To roughly map the protein domains involved in the oligomerization of Otk and Otk2, we generated deletion constructs of Otk and tested them in the same co-immunoprecipitation approach as described above. Both a version of Otk lacking the extracellular domain and a C-terminally truncated Otk protein were able to co-immunoprecipitate full length Otk as well as Otk2 (Figure 2C). This result indicates that the interaction between both Otk proteins may be mediated by their transmembrane domains. It is beyond the scope of this paper to proof this hypothesis, but dimerization via their transmembrane domains has recently been demonstrated for several receptor tyrosine kinases [35].

*Xenopus* PTK7 was demonstrated to interact with Fz7 in Dsh membrane recruitment [23]. To test whether Otk and Otk2 could function as co-receptors with Fz and Fz2 we performed co-immunoprecipitation assays. GFP-tagged Otk or Otk2 were co-overexpressed with Myc-tagged Fz1 or Fz2 in S2r+ cells. Cell lysates were subjected to anti-GFP IP. Immunoblotting with anti-Myc antibody demonstrated that both Otk-GFP and Otk2-GFP robustly co-precipitated with Fz1-Myc and Fz2-Myc (Figure 2D).

### Expression of Off-track and Off-track2 during embryonic and larval development

**Figure 3:**
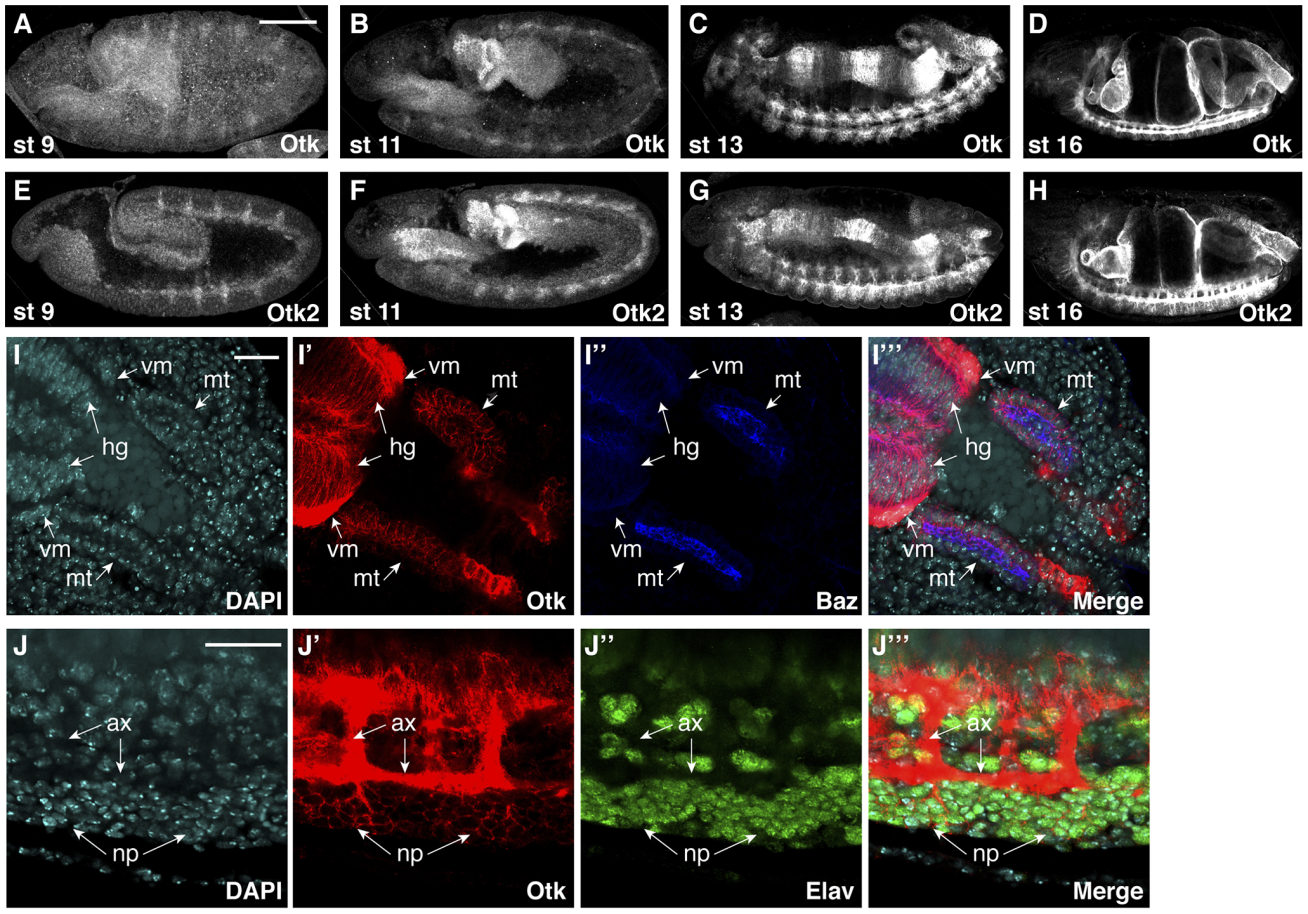
Protein expression of Off-track and Off-track2 in embryos. (A–D) *white* ^−^ embryos were stained with an antibody against the extracellular domain of Otk. (E–H) *white* ^−^ embryos were stained with a peptide antibody raised against Otk2. Stages of embryonic development are indicated. (I–I‴) Detail of the hindgut and Malpighian tubules of a wild type embryo at stage 14 stained for DNA (DAPI, I), Otk (I′) and Baz (I″), merged image in (I‴). Otk is strongly expressed in the visceral mesoderm (vm) surrounding the hindgut and is also expressed in the hindgut epithelium (hg) and in the Malpighian tubules (mt). Note that Otk is localized to the whole plasma membrane and does not show enrichment at the zonula adherens marked by the Baz protein. (J–J‴) Otk is localized to axons and to perikarya of CNS neurons. The image shows a detail from the ventral side of a stage 15 embryo stained for DNA (DAPI, J) Otk (J′) and the neuronal nuclear marker Elav (J″), merged image in (J‴). Note that Otk is strongly enriched on axons (ax) but also stains the plasma membrane of neuronal perikarya (np). In all panels, anterior is to the left. Scale bars in (A–H) = 100 µm, scale bars in (I, J) = 20 µm.

To determine the expression pattern and subcellular localization of Otk and Otk2 during development, polyclonal antibodies were generated against both proteins. The highly dynamic expression pattern of Otk during *Drosophila* embryogenesis has been described before [36] and we were able to confirm the published data with our new antibody against Otk. Interestingly, the expression pattern of Otk2 is essentially identical to that of Otk (Figure 3), indicating that the expression of both genes may be controlled by common regulatory elements.

Otk and Otk2 are first detectable in segmentally repeated stripes in embryos at stage 9–10 (Figure 3A, E). Both proteins are also expressed in the developing central nervous system, the visceral mesoderm, the gut and the Malpighian tubules throughout embryogenesis (Figure 3B–D, F–H). No expression was observed in the epidermis and the salivary glands. The expression data obtained by antibody stainings were confirmed by analysis of reporter lines for both *otk* and *otk2* (Figure S3) and by fluorescent RNA in situ hybridization (FISH; Figure S4).

Otk and Otk2 were expressed in the larval brain (Figure S3H–J), in the leg imaginal discs (Figure S3G), in male and female genital discs (Figure S6) and in developing photoreceptor neurons in third instar eye imaginal discs (Figure S7), but failed to be expressed in the wing imaginal disc (Figure S7)

At the subcellular level, both Otk and Otk2 were localized at the plasma membrane in all tissues analyzed (Figure 3I, J). In the gut and in the Malpighian tubules, both proteins were present on the basolateral plasma membrane domain and showed little co-localization with Bazooka (Baz), which is localized at the zonula adherens (ZA; Figure 3I). Compared to the epithelial cells of the gut, expression levels of Otk and Otk2 were much higher in the visceral mesoderm surrounding the gut (Figure 3I). In the central nervous system, both Otk and Otk2 were present on neuronal processes and on the plasma membrane of the neuronal perikarya (Figure 3J).

FISH analyses using antisense probes against *otk* and *otk2* showed that in embryos at stage 9 the mRNA of both genes was present in segmentally repeated stripes that were in register with the stripes of Wingless expression in the epidermis (Figure S4M–R). Closer inspection of co-stainings using antibodies against Otk and Wg revealed that Otk is in fact expressed in cells located below the Wg expressing cells in the epidermis, mainly in neuroblasts and their progeny, and in the visceral mesoderm (Figure S5). In the gut, Otk and Otk2 are expressed in three domains that overlap with the expression of Wg in the proventriculus, the Malpighian tubules and the region that will form the second midgut constriction (Figure S8) [37], [38].

### Expression of Otk in embryos mutant for different Wnts

**Figure 4:**
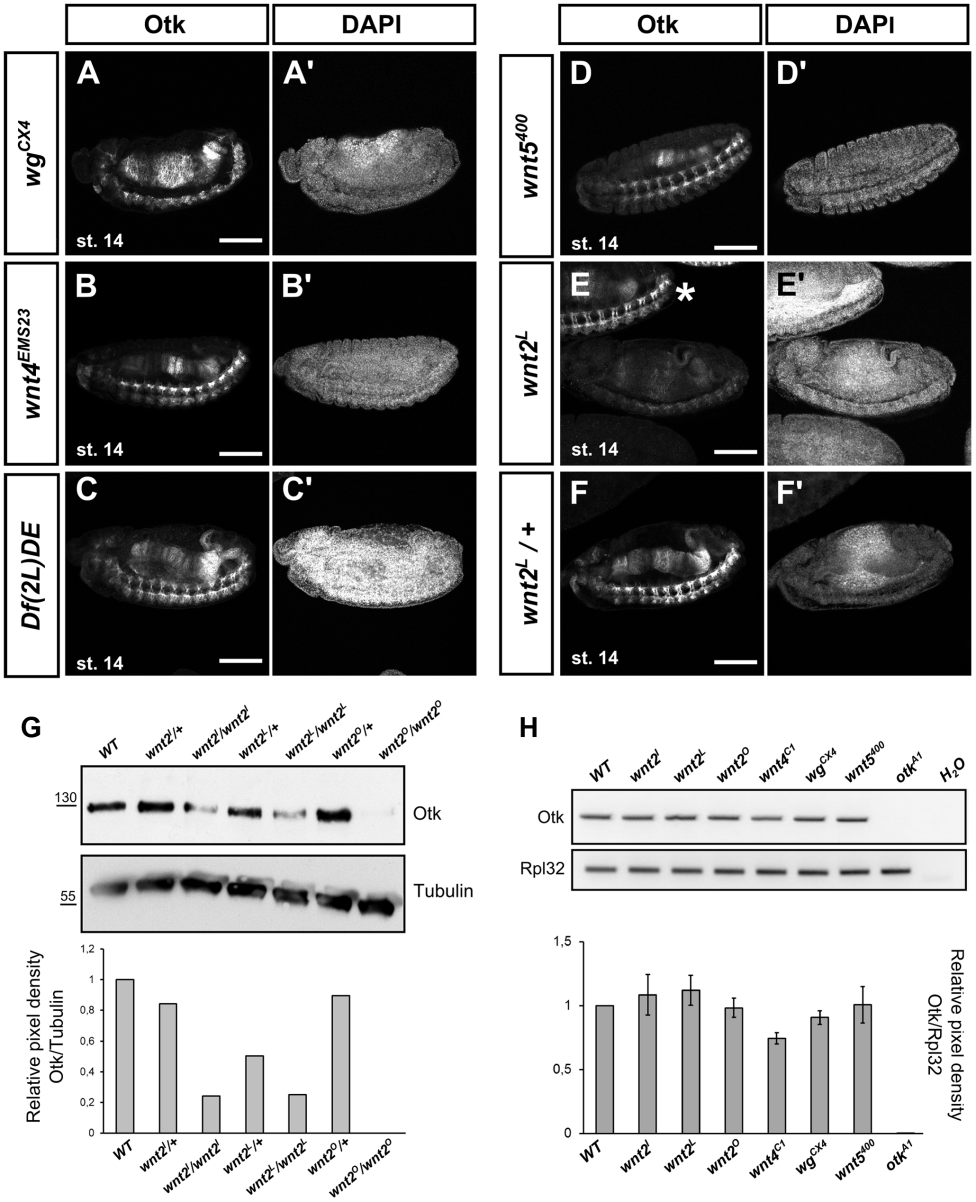
Localization of Otk in embryos homozygous mutant for different Wnt family members. Homozygous mutant embryos of the indicated genotypes were stained against Otk. In embryos homozygous mutant for *wg* (A), *Wnt4* (B), a deficiency removing both *wg* and *Wnt4* (C) and *Wnt5* (D) the level of Otk expression is not changed compared to wild type. (E) Otk expression is strongly reduced in embryos homozygous mutant for *Wnt2^L^*. A heterozygous embryo (asterisk) at the same developmental stage in the top part of panel (E) shows wild type levels of Otk expression. A heterozygous *Wnt2^L^* mutant embryo is shown as control (F). The DAPI staining of the respective embryos is shown in panels (A′–F′). Anterior is to the left. Scale bars = 100 µm. (G) Western blot against Otk of embryonic extracts of the indicated genotypes. Anti tubulin antibody was used as loading control. Band intensity of the Otk blot shown at the top normalized to tubulin levels is quantified at the bottom. (H) RT-PCR analysis of *otk* expression in embryos homozygous mutant for different Wnts. Embryos homozygous mutant for *otk^A1^* were used as negative control. Rpl32 was used as control for the efficiency of the RT-PCR. Otk band intensities normalized to Rpl32 band intensities are quantified at the bottom (n = 2 independent experiments).

To determine whether Otk expression itself might be a target of Wnt signaling, we compared Otk expression in embryos homozygous for mutations in different Wnt genes (Figure 4). Interestingly, Otk does not appear to be regulated by Wg, Wnt4 or Wnt5, since both the segmental localization in early embryos as well as the localization in the developing gut and nervous system were not affected in the respective mutants (Figure 4A–D). By contrast, overall Otk expression levels were strongly reduced in embryos homozygous mutant for *Wnt2* (Figure 4E, F). The reduction of Otk protein levels in *Wnt2* homozygous mutant embryos was also detectable by Western blot of embryonic lysates (Figure 4G). To determine whether Wnt2 controls the transcription of *otk*, we performed semiquantitative RT-PCR analysis. The levels of *otk* transcripts were unaffected by mutation of *Wnt2* (Figure 4H), pointing to posttranscriptional regulation of Otk protein levels by Wnt2. We also tested whether Wnt2 affects the stability of Otk2 in embryos, but levels of Otk2 were unaffected by loss of Wnt2 (Figure S9). To test whether the expression pattern of Wnt2 was compatible with its function in regulating embryonic levels of Otk, we analyzed Wnt2 expression by whole mount in situ hybridization. As described before [39], Wnt2 was expressed in a dynamic, segmentally repeated pattern during embryogenesis (Figure S10). Taking into account that Wnt proteins can spread from their source by diffusion, this pattern of Wnt2 expression is consistent with its apparent function in regulating levels of Otk.

### Otk and Otk2 bind to Wnt2

**Figure 5:**
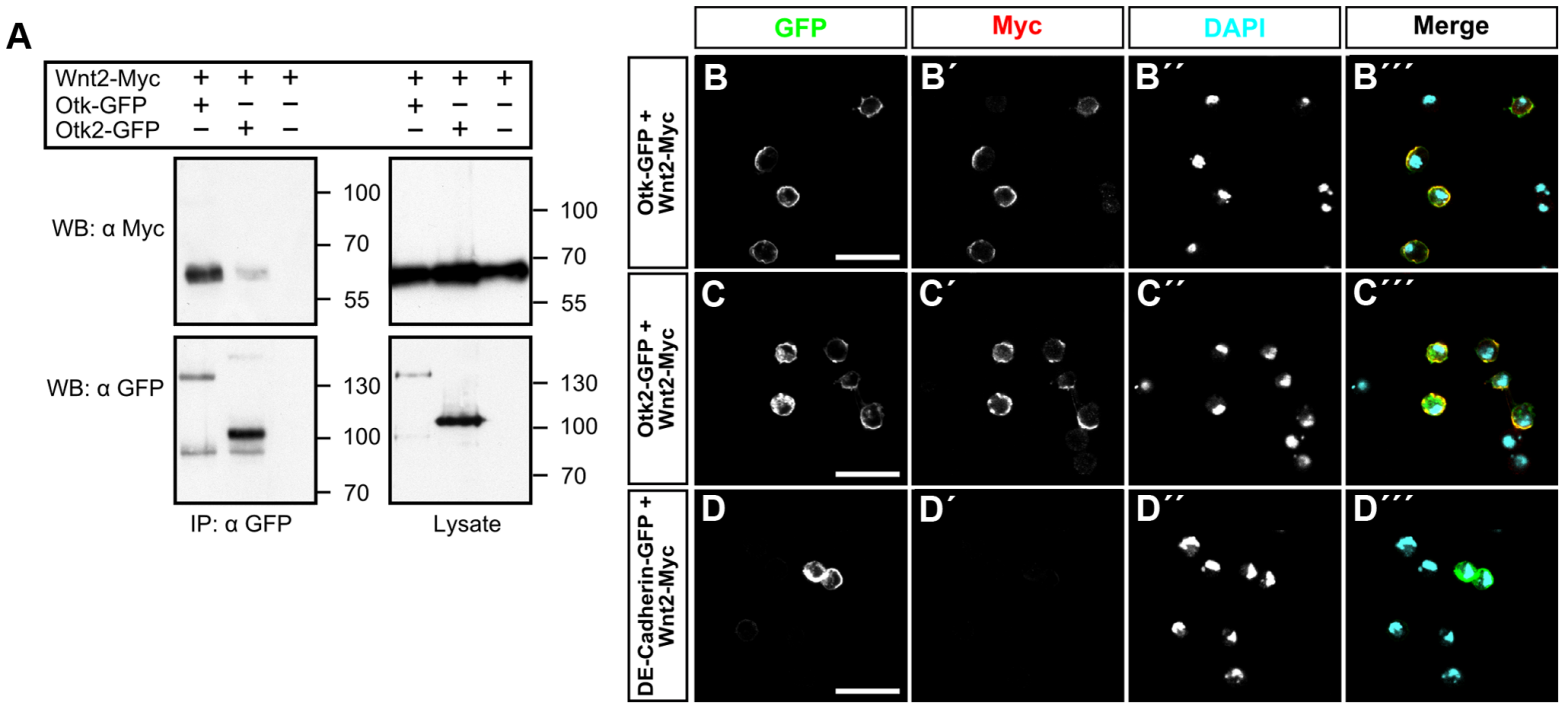
Otk and Otk2 bind to Wnt2. (A) Otk and Otk2 co-precipitate *Drosophila* Wnt2. Otk-GFP or Otk2-GFP and Wnt2-Myc expression vectors were transfected as indicated in Drosophila S2r+ cells. Cell lysates were immunoprecipitated and analyzed by Western Blot with the indicated antibodies. IP, Immunoprecipitation; WB, Western Blot. (B–D) Wnt2 protein binds to S2 cells transfected with Otk-GFP or Otk2-GFP. S2 cells transfected with Otk-GFP (B), Otk2-GFP (C) and DE-Cadherin-GFP (D) were incubated with conditioned medium from S2 cells producing Wnt2-Myc and subsequently stained with anti-Myc antibody. GFP signals are shown in (B–D), Myc signal is shown in (B′–D′), DAPI staining is shown in (B″–D″) and the merged images in (B‴–D‴). Scale bar: 20 µm.

The fact that Otk protein levels are strongly reduced in embryos homozygous mutant for *Wnt2* raises the possibility that Otk and Otk2 may be transmembrane receptors for Wnt2 and that Otk protein stability is regulated by a positive feedback loop. To test this possibility, we performed co-IP experiments between both Otk-GFP and Otk2-GFP and Wnt2-Myc in S2r+ cells. Cells were co-transfected with either Otk-GFP and Wnt2-Myc or with Otk2-GFP and Wnt2-Myc. Cell lysates were precipitated with anti GFP antibody and immunoprecipitates were subjected to Western blot with anti Myc antibody. These experiments revealed that both Otk and Otk2 co-immunoprecipitated with Wnt2 (Figure 5A), consistent with a function for both Otk and Otk2 as receptors for Wnt2.

To further corroborate this finding, we used a cell binding assay described before [2]. In brief, S2 cells lacking Fz and Fz2 expression were transfected with either Otk-GFP or Otk2-GFP and were subsequently incubated with conditioned medium from S2 cells transfected with Wnt2-Myc. Cells were washed and fixed without permeabilization and stained with anti Myc antibody. Only cells expressing Otk-GFP (Figure 5B) or Otk2-GFP (Figure 5C) showed Wnt2-Myc staining on their cell surface, whereas cells without GFP fluorescence failed to stain with the Myc antibody. Control cells transfected with DE-Cadherin-GFP did not show Wnt2-Myc staining on their surface (Figure 5D), demonstrating the specificity of this assay. Together, our results show that both Otk proteins bind to Wnt2.

### Generation of *otk* and *otk2* null alleles

**Figure 6:**
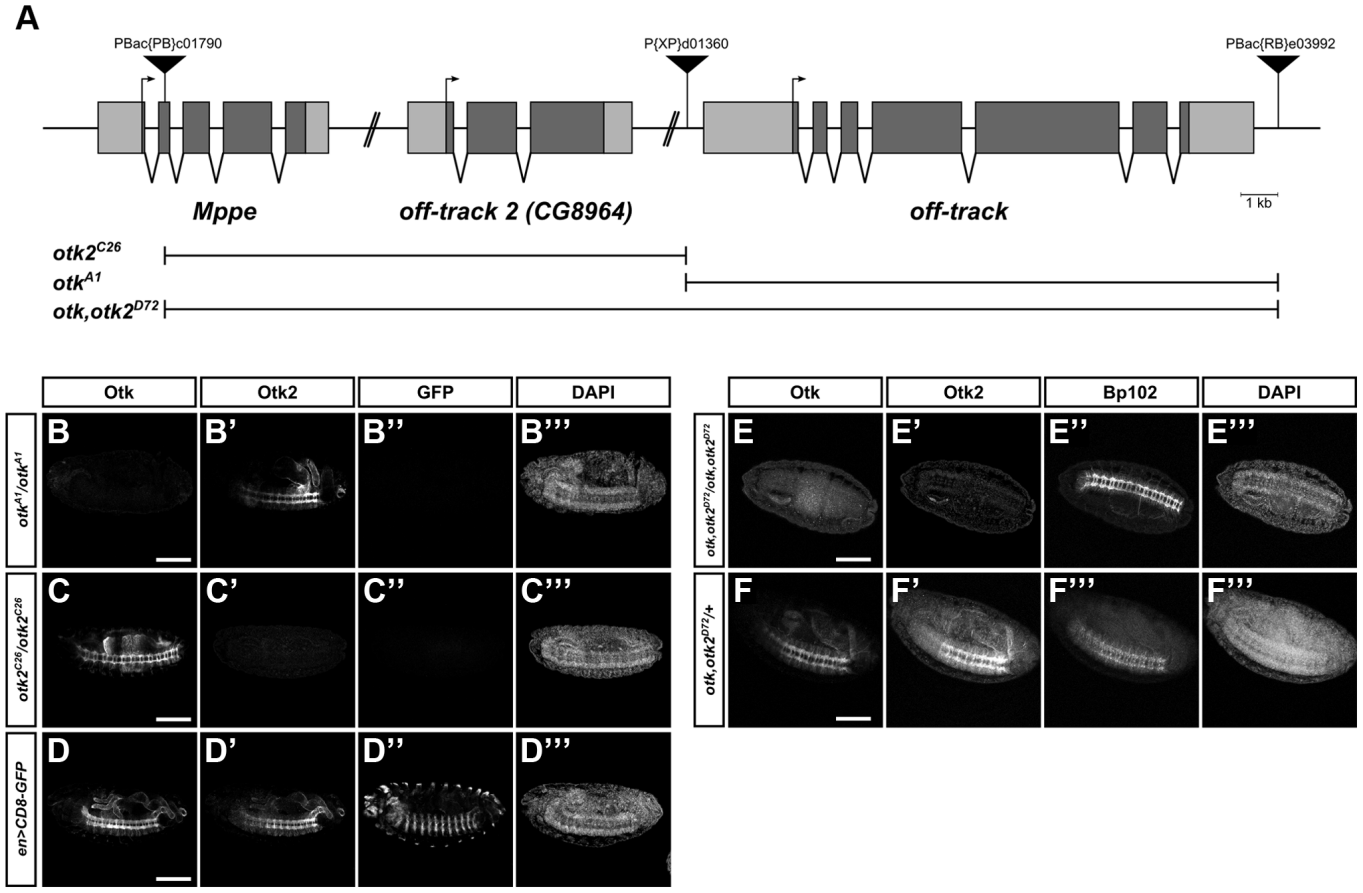
Generation of *otk* and *otk2* null alleles. (A) Overview of the genomic region of *otk*, *otk2* and the neighboring gene *Mppe* located on chromosome 2R. Insertion positions of the three P-element transposons utilized are shown. Null alleles for *otk* and *otk2* alone as well as for both *otk* and *otk2* were generated via FLP/FRT mediated recombination between FRT sites contained in P(XP)d01360 and PBac(RB)e03992 (*otk^A1^*), PBac(PB)c01790 and P(XP)d01360 (*otk2^C26^*) or PBac(PB)c01790 and PBac(RB)e03992 (*otk, otk2^D72^*). (B–F) Verification of the three generated alleles by whole mount immunofluorescent stainings. Homozygous mutant embryos of the indicated genotypes were stained with antibodies against Otk, Otk2, GFP and the CNS axon marker Bp102 as control. (B–D) Wild type embryos expressing CD8-GFP under control of *engrailed::GAL4* were mixed with *otk^A1^* or *otk2^C26^* homozygous mutant embryos prior to fixation and staining. The gain of the confocal microscope was adjusted to the staining intensity of Otk and Otk2 in the wild type embryos and subsequently images of the mutant embryos were taken at exactly the same settings. (B–B‴) In *otk^A1^* homozygous mutant embryos no Otk protein (B) can be detected, but the staining for Otk2 (B′) is normal. (C–C‴) In *otk2^C26^* homozygous mutant embryos no Otk2 protein (C′) can be detected, but the staining for Otk (C) is normal. (D–D‴) wild type control showing normal levels of Otk (D) and Otk2 (D′) expression. The GFP staining of the respective embryos is shown in panels (B″–D″) and the DAPI staining in (B‴–D‴). (E–E‴) In *otk, otk2^D72^* homozygous mutant embryos neither Otk (E) nor Otk2 (E′) protein can be detected. (F–F‴) Heterozygous *otk, otk2^D72^*/+ embryos are shown as control. BP102 staining to label the nervous system is shown in (E″, F″) and DAPI staining in (E‴, F‴). Anterior is to the left. Scale bar = 100 µm.

To further investigate the function of Otk and Otk2, null alleles were generated for both genes as well as a double knock-out. For this purpose the full coding sequences of *otk* and of *otk2* were removed via FLP/FRT-mediated excision [40]. The peculiar chromosomal localization of both genes in tandem offered an easy way to generate a double knock-out for both genes using the same technique as for the *otk* and *otk2* single knock-out. This method utilizes the ability of FLP recombinase to induce recombination between two FRT sites positioned in *trans* on two homologous chromosomes. Three suitable transposon insertion lines containing FRT sites were available from the Harvard stock collection. The P(XP)d01360 element is located upstream of the 5′UTR of *otk* and the PBac(RB)e03992 element is inserted downstream of the 3′UTR of *otk* (Figure 6A). The PBac(PB)c01790 element is located in the second exon of *Mppe*, a gene located upstream of *otk2* and P(XP)d01360 is downstream of the 3′UTR of *otk2* (Figure 6A). FLP recombinase-induced deletion of the genomic region between the FRT sites in P(XP)d01360 and PBac(RB)e03992 was used to remove the coding region of *otk*. Likewise, the genomic region of *otk2* was removed by recombination between the FRT sites located in P(XP)d01360 and PBac(PB)c01790. Finally, excision of the genomic region between the FRT sites in PBac(PB)c01790 and PBac(RB)e03992 deleted the genomic region of both *otk* and *otk2* (Figure 6A).

Parts of the *Mppe* gene upstream of *otk2* were also removed during recombination using PBac(PB)c01790. *Mppe* is not an essential gene and encodes a metallophosphoesterase that functions in Rhodopsin 1 deglycosylation [41]. The following alleles were recovered: *otk^A1^* removes the genomic region of *otk* and *otk2^C26^* the genomic region of *otk2*, while *otk, otk2^D72^* is the double mutant of both genes (Figure 6A). As the three putative deletion lines were all homozygous viable, loss of the respective genes was tested by several methods. Each deletion was verified by PCR on adult genomic DNA (Figure S11A–C) and by Western Blots with protein extracts from homozygous mutant embryos (Figure S11D).

In addition, whole mount immunofluorescent stainings were performed on homozygous mutant embryos (Figure 6B, C, E). These analyses clearly demonstrated that no Otk protein could be detected in *otk^A1^* embryos, while Otk2 localization was normal (Figure 6B). In agreement with this, no Otk2 protein could be detected in *otk2^C26^* embryos, while Otk expression was not changed (Figure 6C). Besides showing that protein-protein interactions between Otk and Otk2 are not required for mutual stabilization of both proteins, these results also demonstrate that both single gene deletions leave intact the genomic regions responsible for regulation of the second homolog, respectively. Finally, neither Otk nor Otk2 could be detected in *otk, otk2^D72^* embryos (Figure 6E). We conclude that the obtained alleles *otk^A1^, otk2^C26^* and *otk, otk2^D72^* are indeed null alleles for the respective genes.

### *otk* and *otk2* are non-essential genes dispensable for planar cell polarity of wings and eyes

Flies homozygous mutant for *otk^A1^* or *otk^C26^* were homozygous viable (Figure S12) and did not show defects in PCP of wings (Figure S13) and eyes (Figure S14). This strongly disagrees with published data on the previously generated *otk^3^* allele, which was reported to be embryonic lethal [12], [31], [32]. We also did not detect any patterning defects of the embryonic cuticle in *otk^A1^*, *otk^C26^* or *otk, otk2^D72^* homozygous mutant embryos, in contrast to an earlier report [12]. Interestingly, also flies homozygous mutant for *otk, otk2^D72^* were viable and did not show any PCP phenotype (Figures S12, S13, S14). Interestingly, we did observe genetic interactions between the *otk, otk2^D72^* double mutant and different alleles of *fz*. Triple homozygosity for mutations in *otk, otk2* and *fz* caused lethality (Table 1). This finding is consistent with a functional interaction between the corresponding proteins, as we already demonstrated by co-IP experiments (Figure 2D).

**Table 1:** The *otk, otk2^D72^* double mutant genetically interacts with *fz*.

Genotype	Phenotype
*otk^A1^/otk^A1^*	viable
*otk2^C26^/otk2^C26^*	viable
*otk,otk2^D72^/otk,otk2^D72^*	viable (male sterile)
*fz^J22^/fz^J22^*	viable with PCP defects
*fz^H51^/fz^H51^*	viable with PCP defects
*Dfz2^C2^/Df(3L)Dfz2*	viable/semilethal
*Dfz2^C2^/Df(3L)469-2*	viable/semilethal
*otk^A1^/otk^A1^; fz^J22^/fz^J22^*	viable with PCP defects
*otk2^C26^/otk2^C26^; fz^J22^/fz^J22^*	viable with PCP defects
*otk,otk2^D72^/+ ; fz^J22^/fz^J22^*	viable with PCP defects
*otk,otk2^D72^/otk,otk2^D72^; fz^J22^/+*	viable
*otk,otk2^D72^/otk,otk2^D72^; fz^J22^/fz^J22^* (zygotic)	**lethal**
*otk,otk2^D72^/otk,otk2^D72^; fz^H51^/fz^H51^* (zygotic)	**lethal**
*otk,otk2^D72^/otk,otk2^D72^; fz^J22^/fz^H51^* (zygotic)	**lethal**
*otk,otk2^D72^/otk,otk2^D72^; Dfz2^C2^/+*	viable (male sterile)
*otk,otk2^D72^/otk,otk2^D72^; Df(3L)469-2/+*	viable (male sterile)

### *otk* and *otk2* function redundantly to ensure male fertility

*otk, otk2^D72^* homozygous mutant males showed fully penetrant sterility. Apparently, both copies of both genes need to be removed to render the males sterile, since transheterozygous *otk*, otk2^D72^/*otk^A1^* or *otk, otk2^D72^/otk2^C26^* males were fertile (Table 2). The sterility of the *otk, otk2^D72^* double mutant was rescued by introduction of a full-length UASp-Otk-GFP transgene expressed under control of the ubiquitous driver *daughterless::Gal4*, demonstrating the specificity of the male sterile phenotype (Table 3). Deletion mutants of Otk lacking either the extracellular or the cytoplasmic domain did not rescue sterility (Table 3), indicating that both domains are required for the function of Otk.

**Table 2:** *otk, otk2^D72^* homozygous mutant males are sterile.

Genotype	Phenotype
*otk,otk2^D72^/otk,otk2^D72^*	Male sterile
*otk^A1^/otk^A1^*	Fertile
*otk2^C26^/otk2^C26^*	Fertile
*otk,otk2^D72^/otk^A1^*	Fertile
*otk,otk2^D72^/otk2^C26^*	Fertile
*otk,otk2^D72^/Df(2R)BSC39* (removes *otk* and *otk2*)	Male Sterile
*otk,otk2^D72^/Df(2R)BSC199* (removes *otk* and *otk2*)	Male sterile
*otk,otk2^D72^/Df(2R)BSC153* (removes only otk)	Fertile
*otk,otk2^D72^/Df(2R)BSC699* (removes only otk2)	Fertile

**Table 3:** Sterility *otk, otk2^D72^* homozygous mutant males can be rescued by an Otk transgene.

Genotype	Phenotype
*otk,otk2^D72^/otk,otk2^D72^*	Male sterile
*otk,otk2^D72^/otk,otk2^D72^; da/UASp::Otk-GFP29*	Fertile
*otk,otk2^D72^/otk,otk2^D72^; da/UASp::OtkDeltaC-GFP14*	Male sterile
*otk,otk2^D72^/otk,otk2^D72^; da/UASp::OtkDeltaEx-GFP20*	Male sterile

To analyze whether sterility is caused by defects in sperm development, testes from males heterozygous and homozygous mutant for *otk, otk2^D72^* were dissected and stained with Vasa, a marker for germline stem cells [42] and Fasciclin III, which marks the hub [43] (Figure S15). The hub consists of non-dividing stromal cells constituting the stromal niche for the germline stem cells and cyst stem cells [44]. Both markers localize normally in testes homozygous mutant for *otk, otk2^D72^* (Figure S15B), indicating that sterility is not caused by any defects in stem cell regulation. Furthermore, co-staining with the DNA marker DAPI revealed that all stages of sperm development, which can be distinguished by their characteristic packaging of the DNA, are present in testes from males homozygous mutant for *otk, otk2^D72^* (Figure S15B″, D′). To further confirm this, testes from males expressing a protamine B-eGFP [45] transgene were analyzed. During *Drosophila* spermatogenesis, histones are replaced by protamines to achieve sufficient chromatin condensation [45]. Indeed, testes from males homozygous mutant for *otk, otk2^D72^* contain all stages of development (Figure S15D), corresponding to testes from heterozygous control males (Figure S15C). Live observation revealed that mature sperm from homozygous mutant males is motile (data not shown). We conclude that any defects in spermatogenesis or sperm motility are unlikely to account for the observed sterility of *otk, otk2^D72^*/*otk, otk2^D72^* adult males. However, after crossing of homozygous mutant *otk, otk2^D72^* males expressing protamine B-eGFP to *white^−^* females, no sperm could be detected in the female reproductive tract, in contrast to the control group with heterozygous mutant males (data not shown). This finding strongly indicates that male sterility of *otk, otk2^D72^*/*otk, otk2^D72^* animals is caused by a structural or mechanical defect of the male reproductive tract.

### *otk, otk2^D72^* homozygous mutant males cannot transfer sperm due to malformation and obstruction of the ejaculatory duct

*Wnt2* was shown to be expressed in genital discs and to be involved in the attachment of the testes to the developing seminal vesicle as well as subsequent myoblast migration [33]. Loss of W*nt2* was reported to result in male sterility due to defects in male reproductive tract formation [33]. Otk as well as Otk2 are expressed in both female and male genital discs as determined by antibody stainings and reporter expression (Figure S6). Because the testes of *Wnt2* mutant males were reported to show gaps in their muscle layer [33], the muscle sheath of the male genital tract of *otk, otk2^D72^* mutant males was analyzed. In contrast to *Wnt2* mutant male flies, the entire genital tract of *otk, otk2^D72^* mutant males was surrounded by a contiguous muscle sheath (Figure S16) and the filament organization of the single organs did not differ from that of heterozygous mutant control flies (Figure S16). It was recently described that the seminal vesicle and the sperm pump contain multinucleated striated muscles, whereas the paragonia and ejaculatory duct are enclosed by mononucleated striated muscle fibers. In contrast, the testes are encircled by smooth muscle fibers [46]. All of these types of muscle fibers could be identified (Figure S16) and no difference between *otk, otk2^D72^* homo- and heterozygous mutant males was observed.

**Figure 7:**
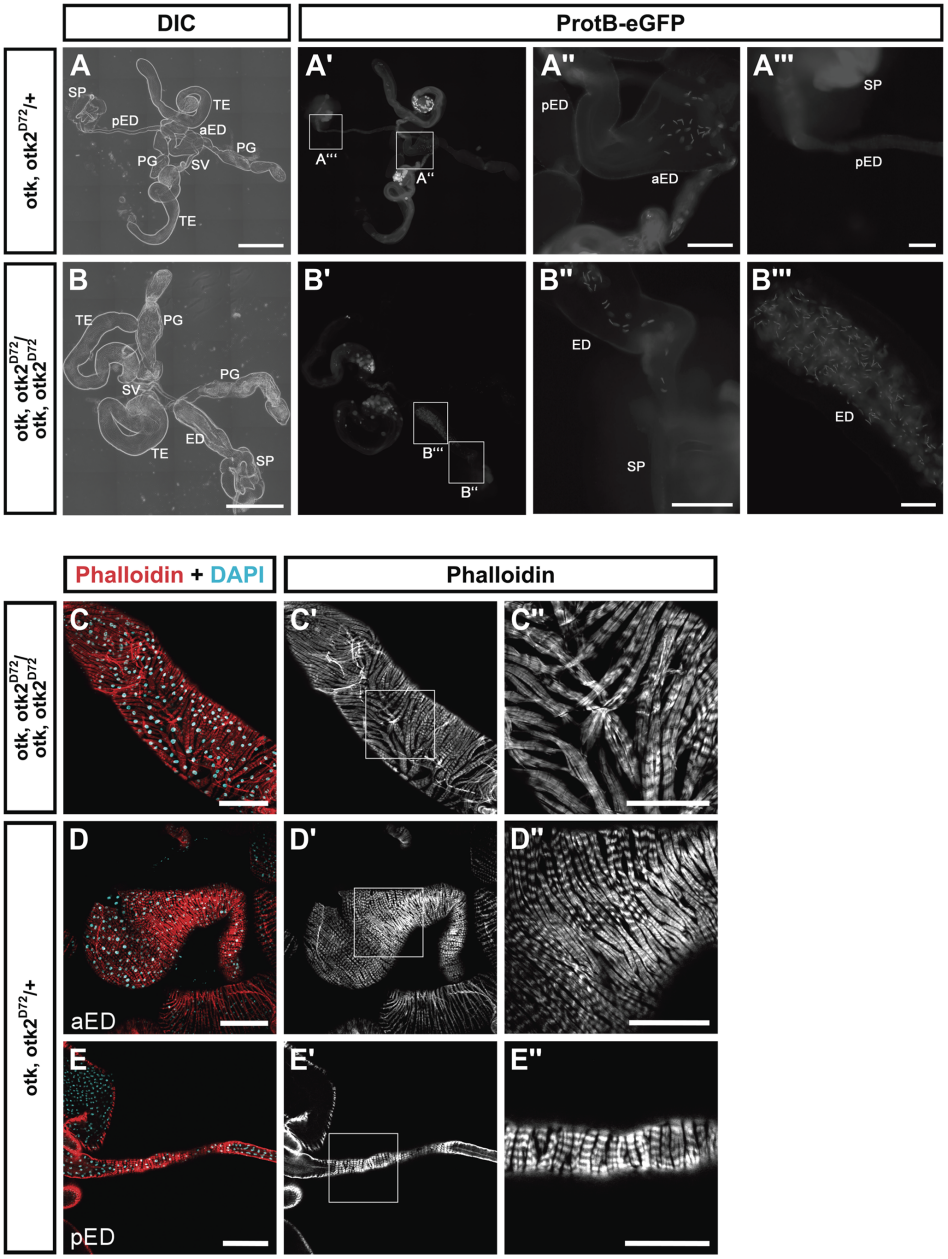
*otk, otk2* loss of function causes malformation and obstruction of the ejaculatory duct. (A) Overview of the reproductive tract of a male heterozygous for *otk, otk2^D72^* and carrying a Protamin B-eGFP transgene. (A″) and (A‴) show higher magnifications of the insets highlighted in (A′). (B) Overview of the reproductive tract of a male homozygous for *otk, otk2^D72^* and carrying a Protamin B-eGFP transgene. Note that the ejaculatory duct is severely shortened and thickened compared to (A). Also note that sperm marked by Protamine B-eGFP (B′) accumulates in the ejaculatory duct of the homozygous mutant male. (B″) and (B‴) show higher magnifications of the insets highlighted in (B′). (C) Disorganization of the muscle sheath of the ejaculatory duct in *otk, otk2* homozygous mutant males. (C″) Enlarged view of the boxed area in (C′). (D, E) The muscle sheath of the anterior (D) and posterior (E) ejaculatory duct from heterozygous control males. (D″, E″) Enlarged views of the boxed areas in (D′, E′). Fluorescent Phalloidin was used to stain F-actin. aED, anterior ejaculatory duct; pED, posterior ejaculatory duct; PG, paragonium (accessory gland); SP, sperm pump; SV, seminal vesicle; TE, testis. Scale bars: A, B = 500 µm, A″, B″ = 100 µm, A‴, B‴ = 50 µm, C–E = 100 µm, C″–E″ = 50 µm.

However, our analyses revealed that the ejaculatory duct of homozygous mutant males was severely malformed (Figure 7). Compared to the heterozygous control (Figure 7A, D, E), in which the posterior ejaculatory duct is a long thin tube, the ejaculatory duct of *otk, otk2^D72^* homozygous mutant males was much shorter and the posterior ejaculatory duct was severely thickened (Figure 7B, C). This phenotype was 100% penetrant (n = 35). The morphology of all the other organs of the reproductive tract was normal (Figure 7B). Consistent with these observations, sperm, which is normally only stored in the seminal vesicle (Figure 7A), accumulated in the ejaculatory duct of *otk, otk2^D72^* homozygous mutant males (Figure 7B), pointing either to an obstruction of the lumen of the duct or to a defect in the transport of sperm through the ejaculatory duct lumen. Closer inspection of the ejaculatory duct revealed that its muscle sheath was strongly disorganized in *otk, otk2^D72^* homozygous mutant males (Figure 7C). We inspected the reproductive tract of *Wnt2^L^/Wnt2^O^* transgeterozygous mutant males in order to check whether their ejaculatory duct was also malformed. This was not the case, whereas we frequently observed missing or incompletely developed testes in the *Wnt2^L^/Wnt2^O^* mutant males (Figure S17). The penetrance of this phenotype was highly allele-dependent and even in the strongest allelic combination, *Wnt2^L^/Wnt2^O^*, the phenotype was variable with about 10% of the transheterozygous males being fertile (Table S1).

### Ubiquitous overexpression of Otk causes malformation of the female oviduct and female sterility

**Figure 8:**
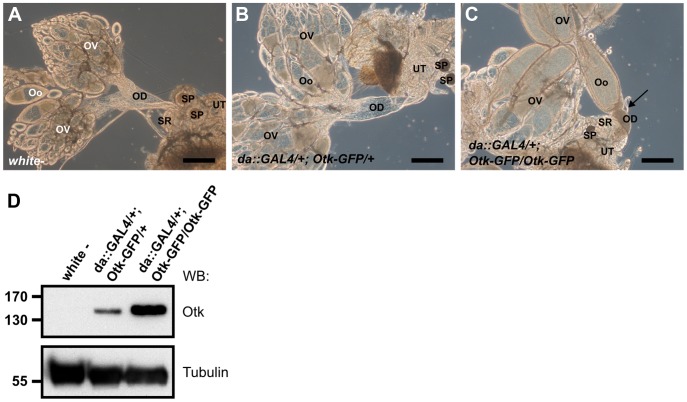
Otk-GFP overexpression causes defective morphogenesis of the oviduct. (A) Genital tract of a wild type female with ovaries (OV), oocytes (Oo), oviduct (OD), seminal receptacle (SR), spermatheca (SP) and uterus (UT). (B) At low levels of Otk-GFP overexpression the female genital tract shows wild type morphology. (C) Strong overexpression of Otk-GFP causes malformation and congestion of the oviduct (arrow) and blocks egg deposition. (D) Western blot of head extracts of the indicated genotypes. Scale bars = 200 µm.

To test whether overexpression of Otk had any effect on development, we ubiquitously overexpressed Otk using the *daughterless::GAL4* driver line. Low level overexpression of Otk had no effect on viability and fertility and the corresponding ovaries (Figure 8B) were indistinguishable from wild type ovaries (Figure 8A). By contrast, flies strongly overexpressing Otk (see Figure 8D for quantitation) were viable, but female sterile. Inspection of the ovaries of these flies revealed that all stages of oogenesis were present (Figure 8C). However, mature eggs were never deposited by the females due to malformation and obstruction of the oviduct (Figure 8C). Together, our data reveal a specific function for Otk and Otk2 in the sexually dimorphic morphogenesis of the reproductive tract in both males and females.

## Discussion

Vertebrate PTK7 is by all commonly accepted criteria a bona fide regulator of PCP that functions in the Wnt signaling pathway as a Wnt co-receptor for canonical Wnt3A and Wnt8 in *Xenopus* [12]. However, it has not been clearly established whether PTK7 promotes signaling by these canonical Wnts or rather functions as an inhibitor of canonical Wnt signaling, which may be essential for activation of the non-canonical PCP branch of Wnt signaling [12], [22], [25], [26]. Here, we investigated whether PCP in *Drosophila* also requires a homolog of PTK7 and if so, whether it functions in a similar manner as vertebrate PTK7.

Our analysis of the function of PTK7 in *Drosophila* revealed several unexpected results. We showed that *otk* is not the single ortholog of PTK7 in the fly genome, as has been previously proposed [12], [18], [31], [32]. In addition to *otk* we identified *otk2* as a second paralog of PTK7 that is most likely the result of a tandem gene duplication that occurred in a common ancestor of all *Drosophila* species with known genome sequences, but not in other arthropods. We furthermore showed that animals homozygous for the null allele *otk^A1^* are viable and fertile, in contrast to the *otk^3^* allele, which was reported to be embryonic lethal [12], [31], [32]. We also did not observe any patterning defects in the embryonic cuticles of homozygous *otk^A1^* null mutant embryos, in contrast to a recently published report using the *otk^3^* allele [12]. None of the previous reports using the *otk^3^* allele showed a rescue of the lethality by a wild type *otk* transgene, which makes it likely that both the lethality and the reported cuticular phenotypes of *otk^3^* are due to a lethal second site mutation on the same chromosome. In a recent paper from the Tolwinski lab [12] it was proposed that Otk may function as a coreceptor for Wnt4 required for proper cuticular patterning. However, we showed that neither Otk nor Otk2 are expressed in the embryonic epidermis, which strongly argues against this hypothesis. We also did not observe any cuticular patterning defects upon ubiquitous overexpression of Otk, as claimed recently [12]. The cuticular patterning defects upon co-overexpression of Otk and Wnt4 shown in the same paper are in fact very similar to those reported for overexpression of Wnt4 alone [47], calling into question any functional interaction between Wnt4 and Otk. We did not try to thoroughly re-examine the occurrence of axon guidance defects as reported for the *otk^3^* allele [31], [32] in the *otk^A1^* null mutant, but until this has been done there remains the possibility that the reported axon guidance phenotypes are also due to the second site lethal mutation on the *otk^3^* mutant chromosome.

The existence of *otk2* as a second gene closely related to PTK7 offered the possibility that *otk2* rather than *otk* is the functional ortholog of PTK7 in the *Drosophila* genome or that *otk* and *otk2* function redundantly. However, like for the *otk^A1^* null mutation, flies homozygous for the *otk2^C26^* null allele were viable and fertile. Even the double mutant *otk, otk2^D72^* was homozygous viable but male sterile and did not display any PCP phenotype in wings, legs and eyes. Both *otk2^C26^* and *otk, otk2^D72^* also remove the *Mppe* locus, which encodes a metallophosphoesterase required for deglycosylation of rhodopsin [41]. However, we can exclude the possibility that *Mppe* is responsible for the male sterile phenotype, because we can rescue sterility of the *otk, otk2^D72^* double mutant by expression of Otk-GFP, and because a null mutation of *Mppe* is homozygous viable and fertile [41]. Thus, our data show that *otk* and *otk2* function redundantly and are required for male fertility, but not for PCP signaling in wings, eyes and the adult cuticle.

How is a function for both *otk/otk2* genes in male fertility compatible with the proposed function of their vertebrate homolog PTK7 in Wnt signaling? It was shown that flies homozygous for a null mutation in *Drosophila* Wnt2 are viable but male sterile [33]. We found that Wnt2 can form a complex with Otk and Otk2 upon co-expression in S2 cells. Moreover, expression of Otk in embryos is strongly reduced in *Wnt2* mutant animals, but appears to be independent of Wg, Wnt4 and Wnt5. We showed that Wnt2 stabilizes Otk at the posttranscriptional level, but we currently can only speculate about the mechanism responsible for this effect. In analogy to LRP6, another important Wnt co-receptor, Wnt2 binding to Otk may induce the phosphorylation of the Otk cytoplasmic tail by a cytoplasmic protein kinase, e. g. Src [16], thus stabilizing Otk in the membrane [48]. Although the expression patterns of Otk and Otk2 are distinct from that of Wnt2 in the embryo (see Figures 3 and S10), Wnt2 may reach areas of Otk and Otk2 expression by diffusion. In the case of tracheal development in the embryo, where Wnt2 functions redundantly with Wg, it has been demonstrated that Wnt2 can diffuse and influence cells in which it is not expressed [49]. Interestingly, the function of Wnt2 in tracheal development depends on Fz and Fz2. This is consistent with our hypothesis that Otk and Otk2 may function as co-receptors for Wnt2, because we also showed that Otk and Otk2 can form protein complexes with Fz and Fz2.

Wnt2 mutant male flies show defects in the muscle sheath surrounding the testis and lack pigment cells associated with the muscle sheath, which is likely to render the male reproductive tract nonfunctional [33]. Males homozygous mutant for *otk, otk2^D72^* do not show exactly the same phenotype but do show an irregular architecture of the muscle sheath surrounding the ejaculatory duct. We indeed found that defective morphogenesis and obstruction of the ejaculatory duct is responsible for the sterility of *otk, otk2^D72^* males. How exactly *otk* together with *otk2* affects the morphogenesis of the ejaculatory duct is unclear at present and very little is known about the development of this region of the genital tract in wild type flies. However, it appears possible that the failure of the posterior ejaculatory duct to elongate and reduce its diameter may be caused by defects in convergent extension movements of the ejaculatory duct cells in *otk, otk2^D72^* males. If that were true, it would be the first example of a component of Wnt signaling regulating convergent extension movements in *Drosophila*. Earlier studies showed that the ejaculatory duct develops from the male genital disc [50] and that the muscle sheath surrounding the testis and the ejaculatory duct develops from adepithelial muscle precursor cells attached to the genital disc [33], [46]. The primary defect presumably causing sterility in Wnt2 mutant males is the defective migration of muscle precursor cells from the genital disc to the testis and the failure to induce pigment cells covering the testis [33]. When we analyzed the morphology of the ejaculatory duct and its muscle sheath in Wnt2 mutant animals we did not detect any obvious abnormalities. Instead, we found that in the strongest allelic combination *Wnt2^O^/Wnt2^L^* the testes were frequently missing or severely reduced in size. However, this phenotype was quite variable and some *Wnt2^O^/Wnt2^L^* males were in fact fertile. Other combinations of *Wnt2* alleles were fully fertile, demonstrating that Wnt2 is not essential for male fertility. Despite of these apparent phenotypic differences between the *Wnt2* mutant and the *otk, otk2^D72^* double mutant, Wnt2 may nonetheless be one of the ligands for the Otk receptors relevant for assuring male fertility. In mammals, several Wnts are involved in the development of the female reproductive tract, including Wnt4, Wnt5a, Wnt7a and Wnt9b [51]. Intriguingly, male and female mice mutant for Wnt7a, the closest homolog of *Drosophila* Wnt2 in mammals, are sterile due to defective morphogenesis of the genital tract. In Wnt7a mutant males the Müllerian duct fails to regress and this leads to a block of sperm passage in the vas deferens, which cannot connect properly at its distal end [34]. In Wnt7a mutant females, morphogenesis of the Müllerian duct derivatives, the oviduct and the uterus, is impaired, which does not allow proper transport and implantation of the ovum [34]. Very interestingly, Wnt7a interacts genetically with Van Gogh-like 2 (Vangl2) in female reproductive tract development [51], [52]. Vangl2 in turn interacts genetically with PTK7 in vertebrate PCP [18], making PTK7 an excellent candidate to test for its involvement in reproductive tract development in mammals.

Together, our findings reveal a redundant function for the transmembrane receptors Otk and Otk2 in male fertility. Our data furthermore support the hypothesis that Otk and Otk2 are co-receptors for Wnt2 and form complexes with Fz and Fz2. These findings raise the question whether mammalian PTK7 interacts with Wnt7a and is required for the function of Wnt7a in reproductive tract development.

## Materials and Methods

### Fly stocks and genetics

The following stocks were used in this study: P(XP)d01360, PBac(PB)c01790, PBac(RB)e03992 (Exelixis collection, Harvard University, MA); *otk* ^CPTI000252^, *CG8964^SH1639^* (Kyoto stock center); Df(3R)BSC39 (#7145), Df(3R)BSC199 (#9626), *wg^CX4^* (#2980), *Wnt4^EMSS23^* (#6650), *Wnt4^C1^* (#6651), Df(2L)DE (#6653), *Wnt2^L^* (#6909), *Wnt2^O^* (#6958), *Wnt2^I^* (#6960), *daughterless*-Gal4 (#5460), y w hs Flp; Sco/CyO (#1929), PhiC31 86FB (#23648) (Bloomington Drosophila stock center, Bloomington, IN; stock numbers given in parentheses). *fz^J22^*, *fz^H51^, fz^P21^* (gifts from Paul Adler); *fz^R52^, fz^R52^Df(3L)Dfz2* (gifts from Ken Cadigan); *Dfz2^C2^*, *Df(3L)469*-2 (gifts from G. Struhl); *Wnt5^400^* [53] and *protamineB-eGFP* [45] were sourced as noted in the references. Null alleles of *otk* and *otk2* were generated by FLP/FRT mediated recombination in trans of the P-element insertions P(XP)d01360, PBac(PB)c01790 and PBac(RB)e03992 [40]. Transgenic fly lines for the constructs UASp::Otk-GFP29, UASp::OtkΔC-GFP14 and UASp::OtkΔEx-GFP20 were generated as described in [54], [55] by injection into an attP landing site at 86FB.

### Molecular biology

The coding regions of full-length or partially deleted versions of *otk*, *otk2*, *fz*, *fz2*, *Wnt2* and *DE-Cadherin* were amplified and the PCR products cloned into pENTR vector using the pENTR Directional TOPO Cloning Kit (Invitrogen, Carlsbad, CA). For expression in S2 and S2r+ cells and for generation of transgenic flies, constructs were recombined into different expression vectors (pAWG, pAWM, pPWG-attB; Murphy lab, Carnegie Institution of Washington, Baltimore, MD) using Gateway technology (Invitrogen).

For expression as a GST-fusion protein, the extracellular domain of Otk corresponding to aa 159–338 was cloned into pGEX-4T-1 (GE Healthcare, Piscataway, NJ) using BamHI and EcoRI restriction sites.

### Antibodies and immunohistochemistry

To raise polyclonal antibodies against Otk, a GST fusion protein corresponding to aa 159–338 of the extracellular domain was purified and injected into guinea pigs (Eurogentec, Seraing, Belgium). The final bleed of one guinea pig was used for all experiments described in this study. Antibodies against Otk2 were generated by immunizing two rabbits with the peptides VELGRMDSTTSEPQLE (aa 93–98, internal fragment) and ESTILEQESQVADDIV (aa 418–433, at C-terminus). Final bleeds were pooled and affinity purified against the C-terminal peptide (Eurogentec, Seraing, Belgium).

For immunohistochemical stainings, the following primary antibodies were used: guinea pig anti Otk, 1∶1000 (this study); rabbit anti Otk2, affinity-purified, 1∶100 (this study); mouse and rabbit anti GFP, 1∶1000 (A11120 and A11121, Invitrogen, Carlsbad, CA); rabbit anti Vasa, 1∶2000, (gift from Ruth Lehmann); mouse anti beta-Galactosidase (JIE7), 1∶20; mouse BP 102, 1∶50; mouse anti c-myc (9E10), 1∶20; mouse anti-Fasciclin III (7G10), 1∶20; mouse anti Wg (4D4), 1∶20 (DSHB, University of Iowa, IA).

Secondary antibodies conjugated to Cy2, Cy3 (Jackson ImmunoResearch Europe, Newmarket, UK) and Alexa Fluor 647 (Invitrogen, Carlsbad, CA) were used at 1∶400 dilution. To visualize F-actin, genital tracts were incubated with 2 U rhodamine-conjugated phalloidin in PBS with 0.1% Tween. DNA was stained with 4′,6-Diamidino-2-Phenylindole (DAPI; Invitrogen, Carlsbad, CA).

Genital tracts were fixed with 4% formaldehyde in phosphate-buffered saline (PBS; pH 7.4). Samples were examined using 25×0,8 NA Zeiss Plan-Neofluar and 63×1,4 NA Zeiss Plan-Apochromat oil immersion objectives on a confocal laser-scanning microscope (Carl Zeiss LSM 510 Meta). Brightfield images were acquired with an AxioImager Z1 upright microscope using 10×0,3 NA Zeiss Plan-Neofluar and 25×0,8 NA Zeiss Plan-Neofluar oil immersion objectives.

### Wnt2 binding assay

S2 cells transfected with GFP tagged Otk, Otk2 or DE-cadherin grown on acid treated coverslips were washed twice in PBS and incubated with 10× concentrated conditioned medium from S2 cells expressing Wnt2-Myc at 4°C for 3 h. After three 10 min washes with cold PBS the cells were fixed in 2% paraformaldehyde for 15 min at room temperature. After three more washes in PBS, fixed cells were incubated with monoclonal mouse anti c-Myc antibody 9E10 at a dilution of 1∶20 overnight at 4°C. After repeated washes in PBS cells were incubated with Cy3-conjugated donkey anti mouse secondary antibody (Jackson ImmunoResearch Europe, Newmarket, UK) at 1∶400, washed in PBS and mounted for microscopy.

### Western blots and Co-Immunoprecipitation (Co-IP)

Protein extraction and Western blots were performed according to standard procedures [56]. For Co-IPs, transiently transfected S2r+ cells were harvested and lysed in 1 ml cold Co-IP lysis buffer (50 mM Tris-Cl pH 7,5, 150 mM NaCl, 1% NP40, 0.2% Na-Deoxycholate with protease inhibitors) by homogenization using a 26 G insulin syringe. Subsequently, the cells were disrupted by sonication with alternating bursts for 10 min. The lysates were then centrifuged and the supernatant was transferred into fresh tubes and pre-cleared with Protein A/G Sepharose beads (BioCat, Heidelberg) for 1 h on a rotator at 4°C. After pre-clearing, 20 µl of each sample were kept as an input control. The antibody-antigen reaction took place overnight on a rotator at 4°C. The beads were subsequently washed and 30 µl of 2× SDS buffer [56] were added to each sample followed by incubation at 95°C for 5 min for protein denaturation. The samples were stored at −20°C or used directly for SDS-PAGE and Western blot.

Antibodies used for Western Blot were guinea pig anti Otk, 1∶1000 (this study); rabbit anti Otk2, affinity-purified, 1∶100 (this study); rabbit anti GFP, 1∶1000 (A11121, Invitrogen); mouse anti c-myc (9E10), 1∶200; mouse anti alpha-Tubulin (12G10), 1∶500 (DSHB). Band intensities of blots were quantified with Photoshop© (Adobe, San Jose, CA).

### Semi-quantitative RT-PCR analysis

Total RNA of 50 embryos was extracted using phenol/chloroform (PeqGold TriFast, Peqlab) or the Qiagen RNeasy Mini kit. cDNA was prepared using random hexamer primers. Gene expression was analysed by semi-quantitative RT-PCR using the GoTaq kit (Promega) and the following primers: Otk (for 5′-CACCCTAAGCTTTGCCAGC-3′ and rev 5′-CTACATGGTCGGGTAAAGTGG-3′) and RpL32 (for 5′-AAGATGACCATCCGCCCAGC-3′ and rev 5′-GTCGATACCCTTGGGCTTGC-3′). Band intensities were quantified with ImageJ.

### Phylogenetic analyses

Phylogenetic analyses were conducted with Mega software version 5 [57] using ClustalW alignment and neighbor-joining method with a gap opening penalty of 60. Dot Plot analysis was performed using DottupP with word size 6 (http://mobyle.pasteur.fr/cgi-bin/portal.py#forms::dottup).

### Fluorescent *in situ* hybridization (FISH)

FISH was performed using the Tyramide Signal Amplification (TSA) Kit (Molecular Probes) according to the protocol provided. For preparation of a Digoxigenin (DIG)-labelled RNA probe, 5 µg of Otk-pOT2 (LP17455; DGRC, Bloomington, IN), Otk2-pFLC-I (RE41180; DGRC) or Wnt2-pFLC-I (RE36604; DGRC) were linearized and purified using the High Pure PCR Product Purification Kit (Roche). In vitro transcription and incorporation of DIG was performed with the DIG RNA labeling kit (SP6/T7) (Roche) and the labeled probe was purified using the RNeasy Plus Mini Kit (Qiagen).

### Mounting of adult wings

Wings were removed from adult flies and dehydrated in 100% isopropanol for at least 5 min. Wings were placed on a glass slide and the isopropanol was allowed to evaporate. Wings were mounted with a small drop of Roti Histokitt (Roth) for microscopic analysis.

### Viability and life span measurements

Viability was determined by aligning about 100 embryos on apple juice agar plates. The embryos were allowed to develop at 25°C and hatching rates were recorded after at least 24 h. Survival curves were recorded as previously described [58]. Cohorts of about 100 males or females were separated two days after hatching and were transferred to fresh vials. Subsequently, flies were transferred to fresh vials every four days and the number of dead flies was recorded.

## Supporting Information

Figure S1 Alignment of the protein sequences of Otk and the gene product of CG8964 (Otk2). (A) Sequence alignment and (B) corresponding dot plot of the protein sequences of Otk and Otk2.
(JPG)

Figure S2 Phylogenetic tree of sequences from different arthropod species homologous to *Drosophila* Otk.
(JPG)

Figure S3 Expression of Otk and Otk2 reporter lines. (A–F) Transgenic embryos expressing a GFP gene trap in the *otk* locus were stained against GFP. Stages of embryonic development are indicated. (G, G′) Leg imaginal disc from L3 larva expressing *otk* GFP gene trap stained against GFP (G), merged image together with DAPI in (G′). (H–J) Brain from L3 larvae expressing *otk* GFP gene trap stained against GFP. (H) Dorsal view of the brain. (I, I′) Magnification of the optic lobes stained for GFP to mark Otk expressing cells (I). Miranda (Mira) and Bazooka (Baz) were co-stained to mark the neuroblasts (I′). (J) Ventral view of the brain. (K–P) Transgenic embryos expressing a lacZ gene trap in the *otk2* locus were stained against beta-galactosidase. Stages of embryonic development are indicated. Anterior is to the left in (A–F) and (K–P). Scale bars: (A–F, H, J, K–P) = 100 µm, (G) = 50 µm, (I) = 20 µm. A homozygous viable GFP enhancer trap line in the *otk* locus and a lacZ enhancer trap line for *otk2* were available. The transposon PBac{602.P.SVS-1}otk^CPTI000252^ (will be called *otk^CPTI000252^* for simplification) is inserted in the first intron of the *otk* locus [position 2R:7,902,551 (+)] and encodes for a GFP gene trap. The line is not a functional protein trap and thus does not provide any insight into the subcellular localization, but is still useful as a reporter line. GFP expression could be observed in a segmental pattern during early embryonic development from stage 9 on (A, B). It is possible that expression starts earlier, but is too weak to be detected. Later on, expression could be observed in the developing nervous system and in the developing gut (C–F). Dissection of wandering L3 larvae revealed expression in the leg imaginal disc (G) as well as in the brain (H–J). In the brain, strong reporter expression was seen in the optic lobes of the central nervous system (H). Co-immunostaining with neuroblast markers showed that the Otk reporter is not expressed in neuroblasts but in the surrounding cells, which are neuroblast progeny (I). Furthermore, expression could be observed in a dorsal structure in the brain, which possibly is the developing mushroom body (J). The transposon P{lacW}l(2)SH1639^SH1639^ (will be called CG8964^SH1639^ for simplification) is inserted in the 5′UTR of the *CG8964* locus (position 2R:7,912,740.7,912,810) and encodes for a lacZ enhancer trap. LacZ expression could be detected in segmental stripes in early embryonic development and later on in the developing gut as well as the developing nervous system (K–P). Hence, the expression is very similar to that observed for *otk^CPTI000252^*.
(JPG)

Figure S4 mRNA expression of *off-track* and *off-track2* during embryonic development. (A–F) Fluorescent *in situ* hybridization (FISH) with antisense probe against *otk* on *white*- embryos. (G–L) FISH with antisense probe against *otk2* on *white*- embryos. (M–O) Embryos hybridized with an antisense probe against *otk* were co-stained against Wingless. (P–R) Embryos hybridized with an antisense probe against *otk2* were co-stained against Wingless. Stages of embryonic development are indicated. Anterior is to the left. Scale bars: (A–R) = 100 µm, (M′–R′) = 50 µm. To confirm the results obtained from the analysis of the *otk* and *otk2* reporter lines and to gain further insight into the mRNA localization, fluorescent *in situ* hybridization (FISH) was performed. Both *otk* and *otk2* mRNA are expressed in segmental stripes in the process of germ band extension (A, G). During germ band retraction, both mRNAs start to be expressed in the developing nervous system (B, H). From stage 12 on, mRNA is also expressed in the developing gut (C–E,. I–L). In late embryos, *otk* and *otk2* mRNA are strongly expressed in the gut as well as the nervous system (E, F, K, L). The expression of both mRNAs in segmentally repeated stripes is reminiscent of the expression of the segment polarity regulator Wingless. To check for a potential co-localization, embryos hybridized with FISH antisense probes were co-stained against Wingless. This clearly demonstrated that both Otk and Otk2 mRNA co-localize with Wingless protein (M–R).
(JPG)

Figure S5 Otk expression overlaps with Wg expression. *white^−^* embryos were stained against Otk and Wg. (A–A″) Otk is expressed in segmental stripes that are in register with Wg expression. (B–B″) Higher magnification reveals expression of Otk in cells located below the Wg expressing cells that represent neuroblasts and their progeny. (C–C″) Otk is highly expressed in the visceral mesoderm (arrowheads). (D–D″) Higher magnification reveals that Otk is expressed along the entire visceral mesoderm and is enriched in a segmental pattern overlapping with Wg. Otk signal is shown in (A–D), Wingless signal in (A′–D′) and the merged images in (A″–D″). Anterior is to the left. Scale bars: A, C = 100 µm, B = 20 µm, D = 50 µm.
(JPG)

Figure S6 Otk and Otk2 are expressed in both male and female genital discs. (A–B″) Genital discs from transgenic flies expressing a GFP gene trap in the *otk* locus were stained against GFP (A, B), Otk2 (A′, B′) and DAPI (A″, B″). Otk staining could not be detected, indicating that *otk^CPTI000252^* is a mutant allele of *otk*. (C–D″) Genital discs from transgenic larvae expressing a lacZ gene trap in the *otk2* locus (C–C″) and from *white^−^* larvae (D–D″) were stained against Otk (C, D), Otk2 (C′, D′) and DAPI (C″, D″). (E–E″) A genital disc stained with secondary antibodies only was used to control for specificity of staining. Scale bars: 50 µm.
(JPG)

Figure S7 Otk and Otk2 expression in eye and wing imaginal discs. (A–A″) Wild type third instar eye-antennal imaginal discs were stained for DAPI (A) Otk (A′) and Otk2 (A″). Both proteins were expressed in developing photoreceptor neurons. (B–B″) Wing imaginal disc from a third instar larva homozygous for the GFP gene trap insertion *otk^CTPI000252^* was stained for DAPI (B), GFP (B′) and Otk (B″). No signal could be detected with both antibodies. While the absence of Otk staining is consistent with *otk^CTPI000252^* being a mutant allele of *otk*, the absence of GFP staining demonstrates that Otk is not expressed in wing imaginal discs.
(JPG)

Figure S8 Off-track is expressed in three regions in the embryonic gut. *white-* embryos (stage 14) were stained against Otk and Wingless. (A–A″) Otk is expressed in the developing gut in three domains that overlap with Wingless expression but are broader than the Wingless stripes. (B–D″) Higher magnification of the anterior (B–B″), median (C–C″) and posterior (D–D″) domain. Embryos were stained against Otk (A–D) and Wingless (A′–D′). The merged images are shown in (A″–D″). Anterior is to the left. Scale bars: (A) 100 µm; (B–D) = 20 µm.
(JPG)

Figure S9 Embryonic expression levels of Otk, but not of Otk2, are dependent on Wnt2. (A–A″″) An embryo heterozygous for *Wnt2^O^* and the balancer chromosome *Cyo, twist::GFP* was stained for Otk (A), Otk2 (A′), GFP (A″) and DAPI (A‴). (B–B″″) A homozygous *Wnt2^O^* mutant embryo (marked by the absence of GFP expression from the balancer) stained with the same antibodies as in (A–A″″) was imaged at the same settings as in (A–A″″). Note that levels of Otk are strongly reduced, whereas Otk2 levels are unaffected. Merged images are shown in (A″″, B″″). Scale bar = 50 µm. Anterior is to the top.
(JPG)

Figure S10 Embryonic expression of Wnt2. Whole mount in situ hybridization reveals the embryonic expression pattern of Wnt2 in a segmentally repeated pattern in the dorsal and ventral epidermis (A–C) and in the developing gonad (arrows in D–F). The signal in the salivary glands (arrowheads in [D, E]) is an artifact. Scale bar = 100 µm.
(JPG)

Figure S11 Generation of *otk* and *otk2* null alleles. (A–C) Verification of the three generated alleles by PCR. First, PCRs were done on the transition from the genomic region to the ends of the P-elements to verify that the inner ends of the P-elements are deleted, while the outer ends are still left after recombination has taken place. Furthermore, PCR was performed with primer pairs that encompass the entire deleted regions. All the resulting PCR bands were purified and sequenced. (A) Verification of the *otk^A1^* deletion by PCR on genomic DNA from adult flies. Lane 1–4: The transition between the ends of the P-elements and the genomic region was amplified. PCR was done on the left (1) and right (2) end of P(XP)d01360 and on the left (3) and right (4) end of PBac(RB)e03992. Missing bands indicate the loss of the inner P-element ends. Lane 5 (asterisk): The deleted site was amplified with primers binding upstream and downstream of the whole deletion in the genomic region. The band obtained was purified and sequenced. Adult fly genomic DNA from P(XP)d01360 (lane 6) and PBac(RB)e03992 (lane 7) was used as control. Missing bands can be explained by the large size of the expected fragments. (B) Verification of the *otk2^C26^* deletion by PCR on genomic DNA from adult flies. Lane 1–4: The transition between the ends of the P-elements and the genomic region was amplified. PCR was done on the left (1) and right (2) end of PBac(PB)c01790 and on the left (3) and right (4) end of P(XP)d01360. Missing bands indicate the loss of the inner P-element ends. Furthermore, the deleted site was amplified with primers binding upstream of the deletion in the genomic region and the residual transposon (lane 5) as well as primers binding in the residual transposon and downstream of the deletion in the genomic region (lane 6). The bands obtained were purified and sequenced. (C) Verification of the *otk, otk2^D72^* deletion by PCR on genomic DNA from adult flies. Lane 1–4: The transition between the ends of the P-elements and the genomic region was amplified. PCR was done on the left (1) and right (2) end of PBac(PB)c01790 and on the left (3) and right (4) end of PBac(RB)e03992. Missing bands indicate the loss of the inner P-element ends. Lane 5: The deleted site was amplified with primers binding upstream and downstream of the whole deletion in the genomic region. The band obtained was purified and sequenced. (D) Western Blots were performed with protein extracts from homozygous mutant embryos and the corresponding original transposon lines as well as chromosomal deficiencies as controls. Western blots were probed with the antibodies shown. Detection with the antibody against the extracellular domain of Otk resulted in a band of approximately 120 kDa, corresponding to full-length Otk. Protein extracts from embryos of the original transposon stocks (lane 1–3) were used as controls. No Otk protein was detected in *otk^A1^* (lane 4) and *otk, otk2^D72^* (lane 5) homozygous mutant embryos as well as in embryos carrying the PBac(RB)e03992 P-element (lane 3). Detection with the peptide antibody against Otk2 resulted in a band of approximately 65 kDa, corresponding to full-length Otk2. No Otk2 protein was detected in *otk2^C26^* (lane 5) and *otk, otk2^D72^* (lane 6) homozygous mutant embryos. An antibody against tubulin was used as loading control.
(JPG)

Figure S12 Novel *otk* and *otk2* loss of function mutants are homozygous viable. Lethality assays were performed with embryos of the indicated genotypes. In case of the double deletion, maternally mutant embryos derived from homozygous mutant *otk, otk2^D72^* mothers and heterozygous fathers were used. Embryos were collected and allowed to develop for two days at 25°C. The number of hatched embryos was determined. The experiment was repeated three times. Error bars represent the standard error of the mean.
(JPG)

Figure S13 Homozygous *otk^A1^* and *otk, otk2^D72^* flies do not display any wing PCP defects. Defects in planar cell polarity (PCP) signaling usually can be recognized by disorganization of wing hairs as well as thorax and leg bristles of adult flies [59], [60]. In contrast, failure in canonical Wingless signaling leads to wing margin defects [61], [62]. In contrast to the function of PTK7 in vertebrate PCP signaling [12], [18], [19], [23], loss of neither *otk* nor *otk2* alone (C, D; data for *otk2* not shown) nor of both *otk* and *otk2* (E) leads to any defects in wing hair orientation compared to *w^−^* (A, A′) and the P-element line P(XP)d01360 (B) as controls. (F) Wings from *dsh1/Y* flies as an example for a characteristic PCP phenotype. The phenotype is unchanged in double mutants for *dsh1* and *otk, otk2^D72^* (G). (A) shows a 10× magnification of an adult wing and the five regions of the wing are indicated by roman numerals. (A′, B–G) display 25× magnifications of region IV of the respective wing. Anterior is up and proximal is to the left.
(JPG)

Figure S14 Eyes of homozygous *otk^A1^* and *otk, otk2^D72^* flies do not display any PCP defects. (A) Schematic representation of the ommatidia structure of an adult eye. The photoreceptors in each ommatidium are arranged in an arrow-like manner with the arrow tips pointing away from the equator of the eye (A, B). This organization is frequently disturbed in PCP mutants [59], [60]. Eyes from adult flies homozygous mutant for *otk^A1^* (C) and *otk, otk2^D72^* (D) do not show any defects compared to wild type (B). n = 30 eyes were analyzed for each genotype. 100% of ommatidia showed a wild type orientation in each genotype.
(JPG)

Figure S15 Spermatogenesis in *otk, otk2^D72^* homozygous mutant males. (A–B‴) Adult testes from males heterozygous (A–A‴) and homozygous mutant (B–B‴) for *otk, otk2^D72^* were stained against Fasciclin III (A, B), which marks the hub (arrowhead in A and B) and terminal epithelium, and against Vasa (A′, B′), a marker for germ line stem cells. DAPI staining is shown in (A″, B″), merged images in (A‴, B‴). (C–D′) Higher magnification of sperm in testes from adult males heterozygous (C, C′) and homozygous mutant (D, D′) for *otk, otk2^D72^* carrying a ProtaminB-eGFP transgene. Scale bars: (A, B) = 100 µm, (C, D) = 50 µm.
(JPG)

Figure S16 Morphology of the muscle sheath of the male reproductive system of homozygous *otk, otk2* mutant animals. The muscle sheaths of the different organs of the male reproductive tract of *otk, otk2^D72^* homozygous mutant males were visualized with Phalloidin for F-actin detection. As described in [46] the testis (A) is surrounded by smooth muscle, whereas the seminal vesicles (B), paragonia (C) and sperm pump (D) are ensheathed by striated musculature. Scale bars: (A–D) = 50 µm; (A′–D′) = 20 µm.
(JPG)

Figure S17 Morphology of the reproductive tract of *Wnt2^O^/Wnt2^L^* transheterozygous males. The Figure shows the variability of the reproductive tract morphology in *Wnt2* null mutant males. Whereas the reproductive tract in (A) completely lacks testes, the reproductive tract in (B) has one intact and one rudimentary testis. aED, anterior ejaculatory duct; pED, posterior ejaculatory duct; PG, paragonium (accessory gland); SP, sperm pump; SV, seminal vesicle; TE, testis; rTE, rudimentary testis. Scale bars = 500 µm.
(JPG)

Table S1 Fertility of transheterozygous *Wnt2* mutant males.
(DOCX)
