# Supplementary figures and images for: The PTK7-Related Transmembrane Proteins Off-track and Off-track 2 Are Co-receptors for Drosophila Wnt2 Required for Male Fertility
Source: PLoS Genet. 2014 Jul 10;10(7):e1004443. doi: 10.1371/journal.pgen.1004443 (PMC4091708; doi:10.1371/journal.pgen.1004443)

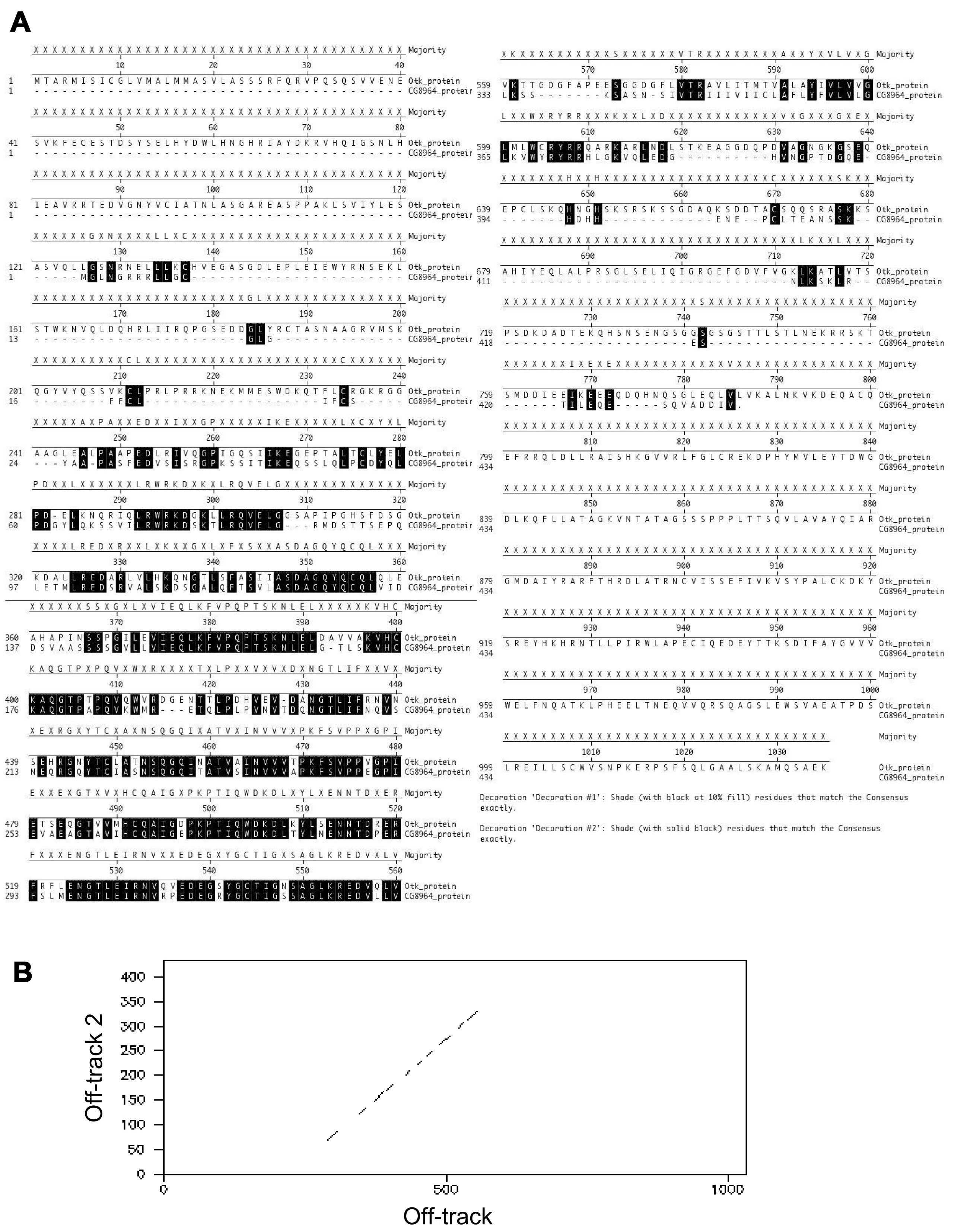

Supplement: Figure S1 — Alignment of the protein sequences of Otk and the gene product of CG8964 (Otk2). (A) Sequence alignment and (B) corresponding dot plot of the protein sequences of Otk and Otk2. (JPG) [file pgen.1004443.s001.jpg]

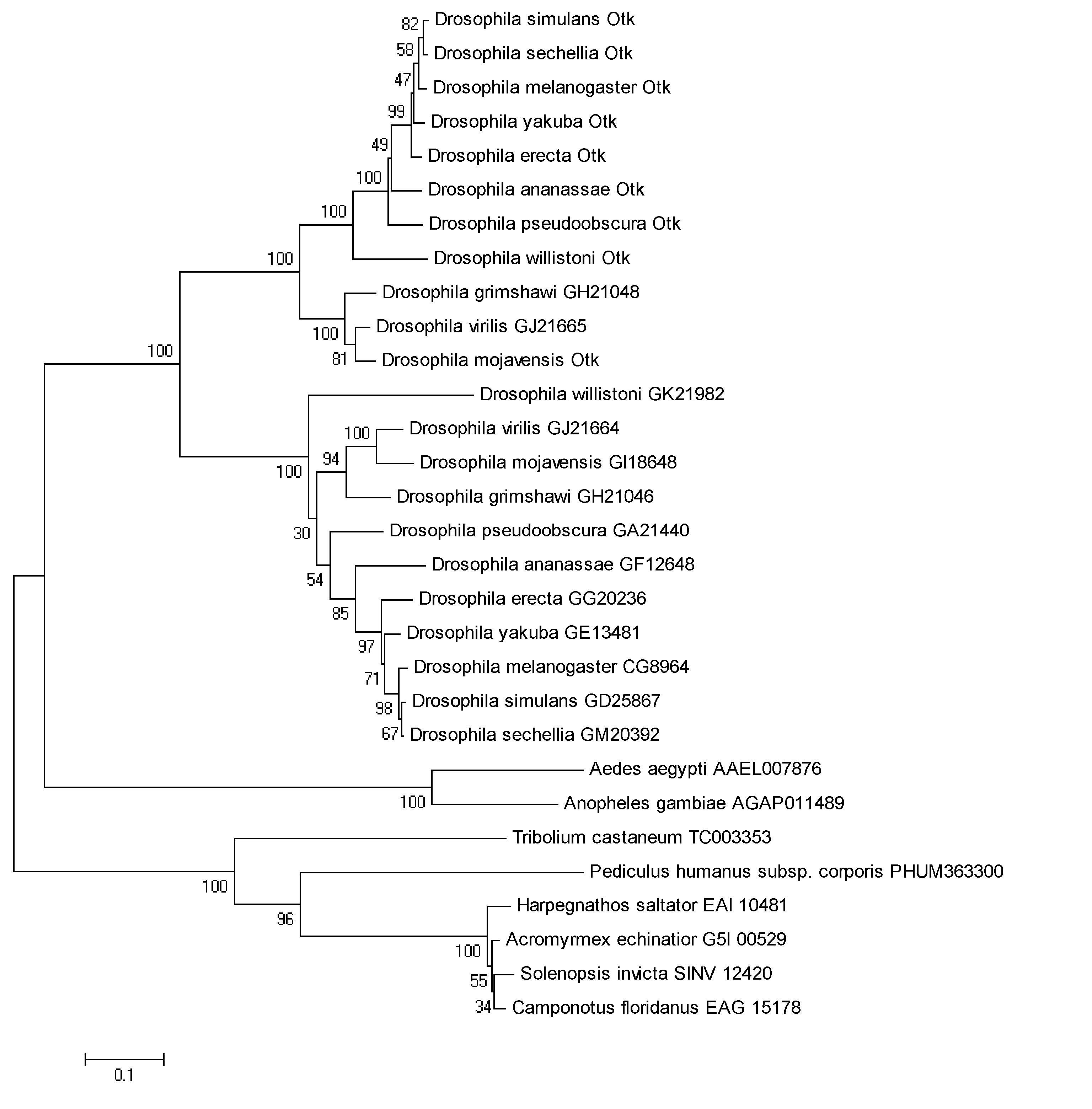

Supplement: Figure S2 — Phylogenetic tree of sequences from different arthropod species homologous to Drosophila Otk. (JPG) [file pgen.1004443.s002.jpg]

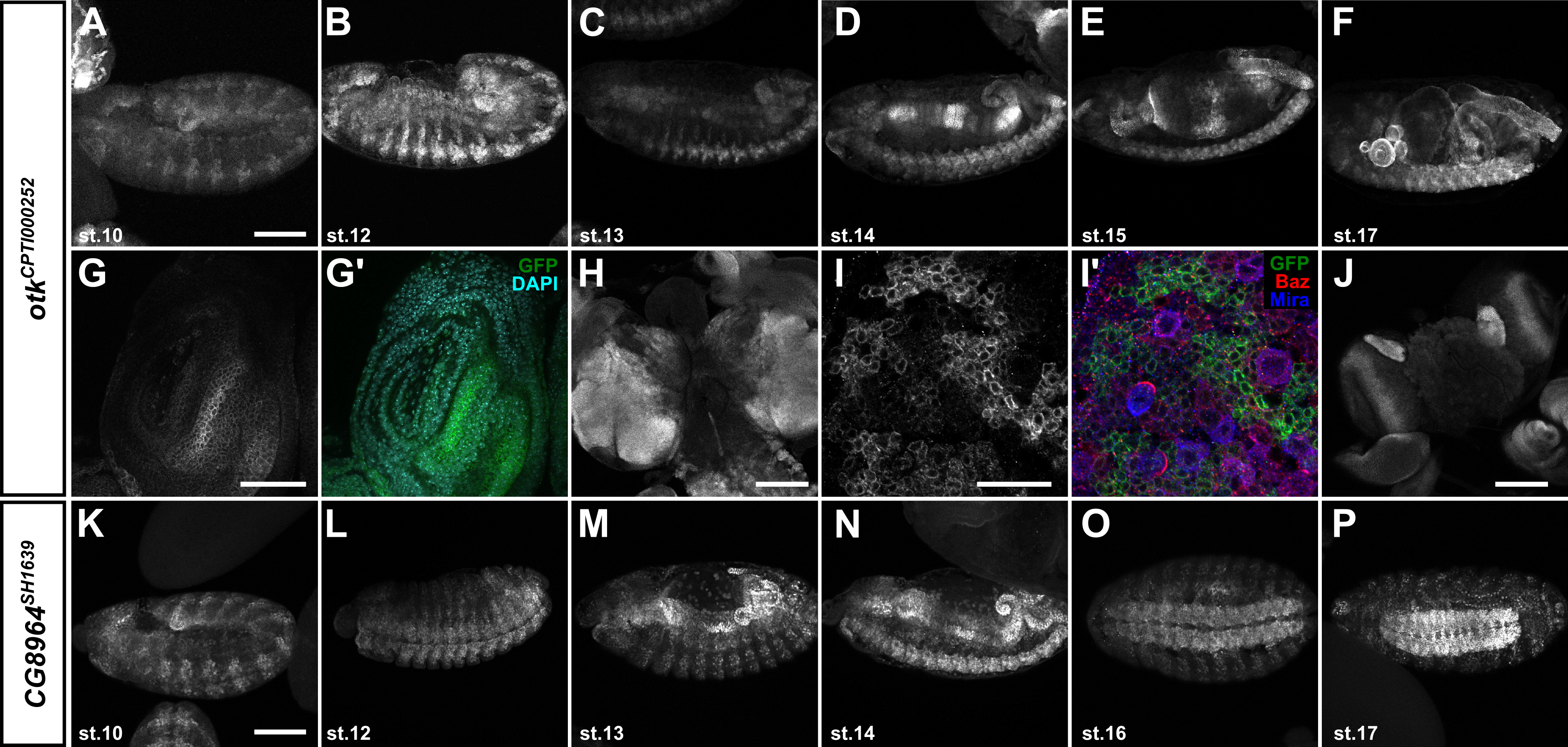

Supplement: Figure S3 — Expression of Otk and Otk2 reporter lines. (A–F) Transgenic embryos expressing a GFP gene trap in the otk locus were stained against GFP. Stages of embryonic development are indicated. (G, G′) Leg imaginal disc from L3 larva expressing otk GFP gene trap stained against GFP (G), merged image together with DAPI in (G′). (H–J) Brain from L3 larvae expressing otk GFP gene trap stained against GFP. (H) Dorsal view of the brain. (I, I′) Magnification of the optic lobes stained for GFP to mark Otk expressing cells (I). Miranda (Mira) and Bazooka (Baz) were co-stained to mark the neuroblasts (I′). (J) Ventral view of the brain. (K–P) Transgenic embryos expressing a lacZ gene trap in the otk2 locus were stained against beta-galactosidase. Stages of embryonic development are indicated. Anterior is to the left in (A–F) and (K–P). Scale bars: (A–F, H, J, K–P) = 100 µm, (G) = 50 µm, (I) = 20 µm. A homozygous viable GFP enhancer trap line in the otk locus and a lacZ enhancer trap line for otk2 were available. The transposon PBac{602.P.SVS-1}otkCPTI000252 (will be called otkCPTI000252 for simplification) is inserted in the first intron of the otk locus [position 2R:7,902,551 (+)] and encodes for a GFP gene trap. The line is not a functional protein trap and thus does not provide any insight into the subcellular localization, but is still useful as a reporter line. GFP expression could be observed in a segmental pattern during early embryonic development from stage 9 on (A, B). It is possible that expression starts earlier, but is too weak to be detected. Later on, expression could be observed in the developing nervous system and in the developing gut (C–F). Dissection of wandering L3 larvae revealed expression in the leg imaginal disc (G) as well as in the brain (H–J). In the brain, strong reporter expression was seen in the optic lobes of the central nervous system (H). Co-immunostaining with neuroblast markers showed that the Otk reporter is not expressed in neuroblasts but in t [file pgen.1004443.s003.jpg]

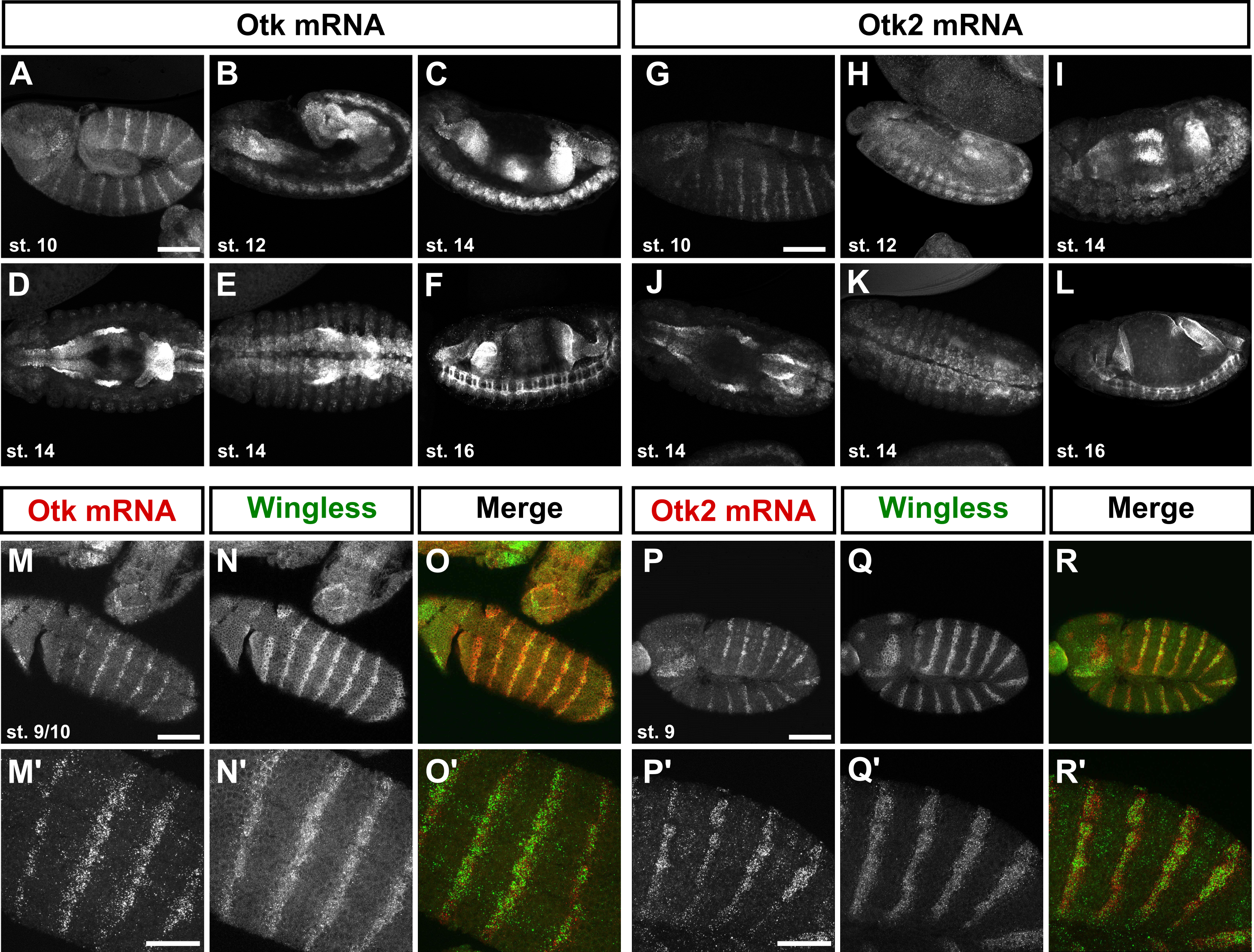

Supplement: Figure S4 — mRNA expression of off-track and off-track2 during embryonic development. (A–F) Fluorescent in situ hybridization (FISH) with antisense probe against otk on white- embryos. (G–L) FISH with antisense probe against otk2 on white- embryos. (M–O) Embryos hybridized with an antisense probe against otk were co-stained against Wingless. (P–R) Embryos hybridized with an antisense probe against otk2 were co-stained against Wingless. Stages of embryonic development are indicated. Anterior is to the left. Scale bars: (A–R) = 100 µm, (M′–R′) = 50 µm. To confirm the results obtained from the analysis of the otk and otk2 reporter lines and to gain further insight into the mRNA localization, fluorescent in situ hybridization (FISH) was performed. Both otk and otk2 mRNA are expressed in segmental stripes in the process of germ band extension (A, G). During germ band retraction, both mRNAs start to be expressed in the developing nervous system (B, H). From stage 12 on, mRNA is also expressed in the developing gut (C–E,. I–L). In late embryos, otk and otk2 mRNA are strongly expressed in the gut as well as the nervous system (E, F, K, L). The expression of both mRNAs in segmentally repeated stripes is reminiscent of the expression of the segment polarity regulator Wingless. To check for a potential co-localization, embryos hybridized with FISH antisense probes were co-stained against Wingless. This clearly demonstrated that both Otk and Otk2 mRNA co-localize with Wingless protein (M–R). (JPG) [file pgen.1004443.s004.jpg]

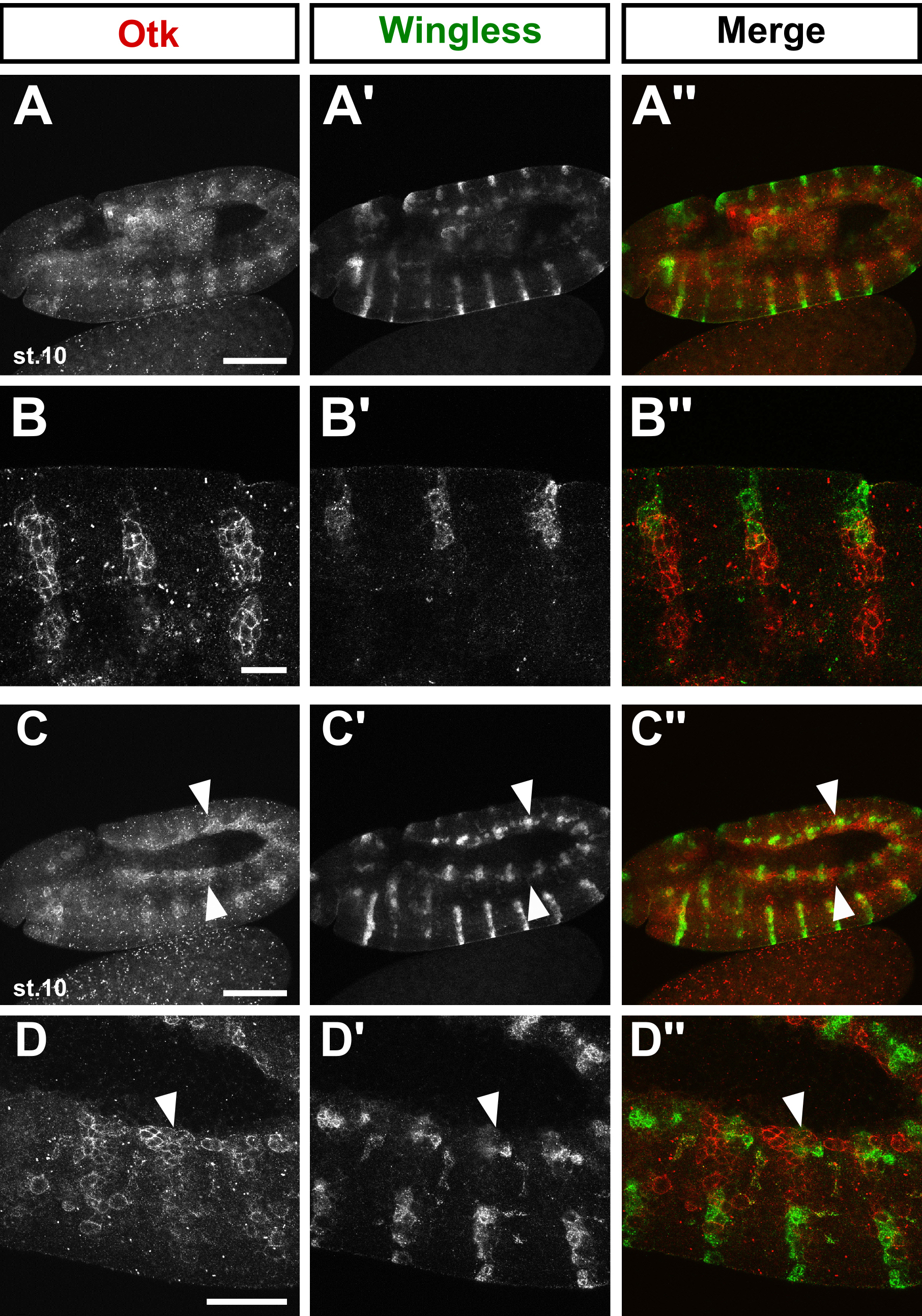

Supplement: Figure S5 — Otk expression overlaps with Wg expression. white− embryos were stained against Otk and Wg. (A–A″) Otk is expressed in segmental stripes that are in register with Wg expression. (B–B″) Higher magnification reveals expression of Otk in cells located below the Wg expressing cells that represent neuroblasts and their progeny. (C–C″) Otk is highly expressed in the visceral mesoderm (arrowheads). (D–D″) Higher magnification reveals that Otk is expressed along the entire visceral mesoderm and is enriched in a segmental pattern overlapping with Wg. Otk signal is shown in (A–D), Wingless signal in (A′–D′) and the merged images in (A″–D″). Anterior is to the left. Scale bars: A, C = 100 µm, B = 20 µm, D = 50 µm. (JPG) [file pgen.1004443.s005.jpg]

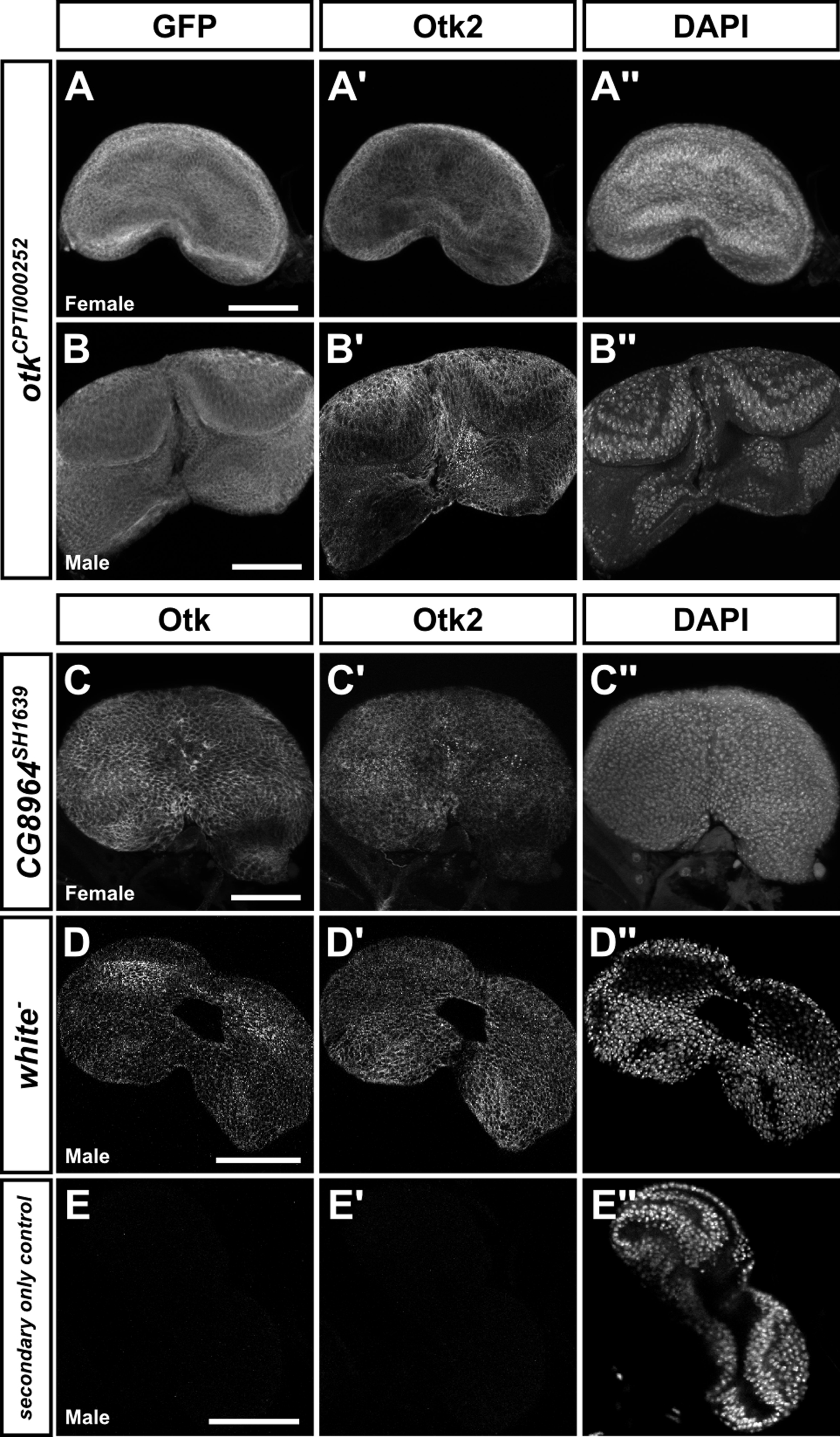

Supplement: Figure S6 — Otk and Otk2 are expressed in both male and female genital discs. (A–B″) Genital discs from transgenic flies expressing a GFP gene trap in the otk locus were stained against GFP (A, B), Otk2 (A′, B′) and DAPI (A″, B″). Otk staining could not be detected, indicating that otkCPTI000252 is a mutant allele of otk. (C–D″) Genital discs from transgenic larvae expressing a lacZ gene trap in the otk2 locus (C–C″) and from white− larvae (D–D″) were stained against Otk (C, D), Otk2 (C′, D′) and DAPI (C″, D″). (E–E″) A genital disc stained with secondary antibodies only was used to control for specificity of staining. Scale bars: 50 µm. (JPG) [file pgen.1004443.s006.jpg]

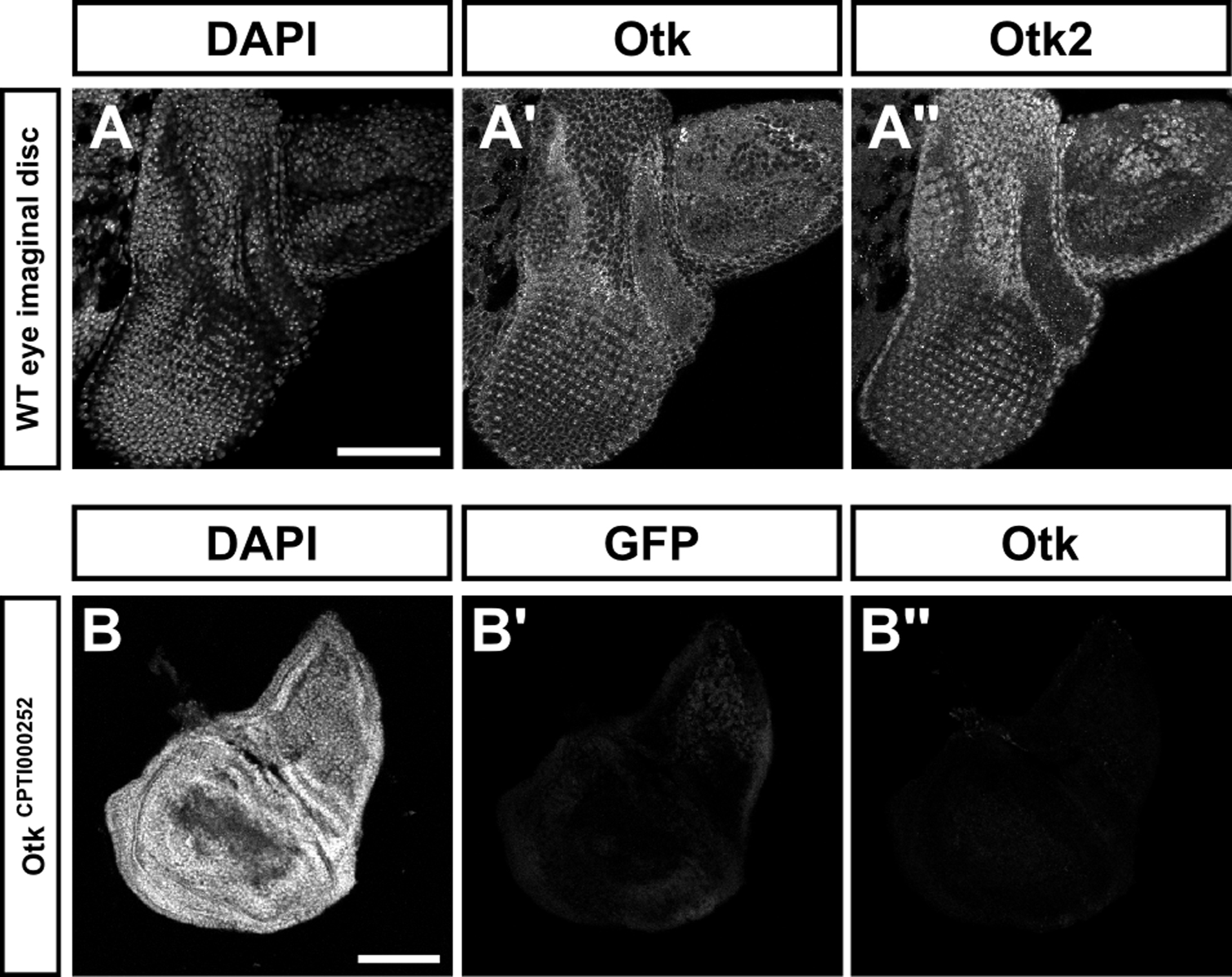

Supplement: Figure S7 — Otk and Otk2 expression in eye and wing imaginal discs. (A–A″) Wild type third instar eye-antennal imaginal discs were stained for DAPI (A) Otk (A′) and Otk2 (A″). Both proteins were expressed in developing photoreceptor neurons. (B–B″) Wing imaginal disc from a third instar larva homozygous for the GFP gene trap insertion otkCTPI000252 was stained for DAPI (B), GFP (B′) and Otk (B″). No signal could be detected with both antibodies. While the absence of Otk staining is consistent with otkCTPI000252 being a mutant allele of otk, the absence of GFP staining demonstrates that Otk is not expressed in wing imaginal discs. (JPG) [file pgen.1004443.s007.jpg]

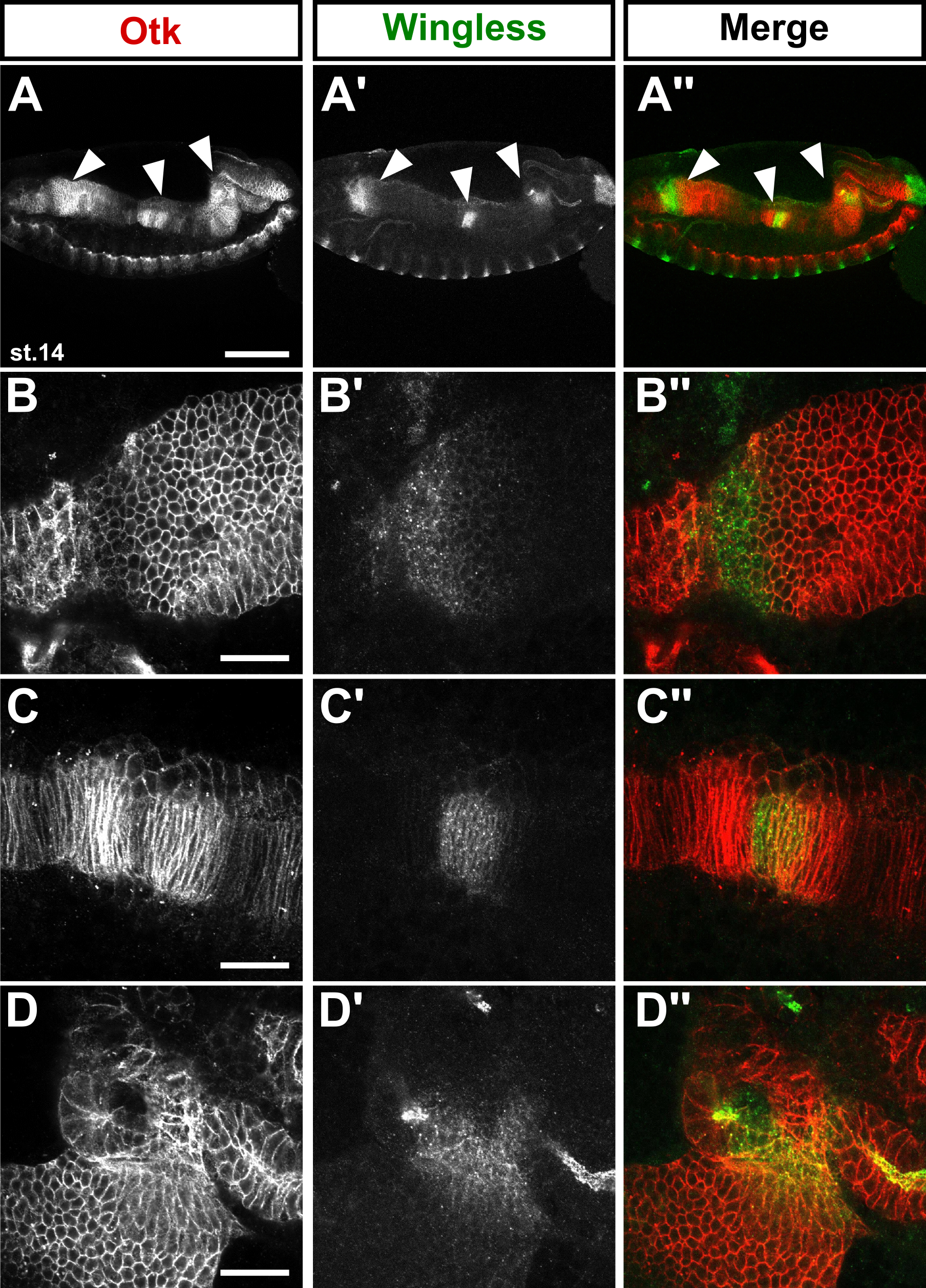

Supplement: Figure S8 — Off-track is expressed in three regions in the embryonic gut. white- embryos (stage 14) were stained against Otk and Wingless. (A–A″) Otk is expressed in the developing gut in three domains that overlap with Wingless expression but are broader than the Wingless stripes. (B–D″) Higher magnification of the anterior (B–B″), median (C–C″) and posterior (D–D″) domain. Embryos were stained against Otk (A–D) and Wingless (A′–D′). The merged images are shown in (A″–D″). Anterior is to the left. Scale bars: (A) 100 µm; (B–D) = 20 µm. (JPG) [file pgen.1004443.s008.jpg]

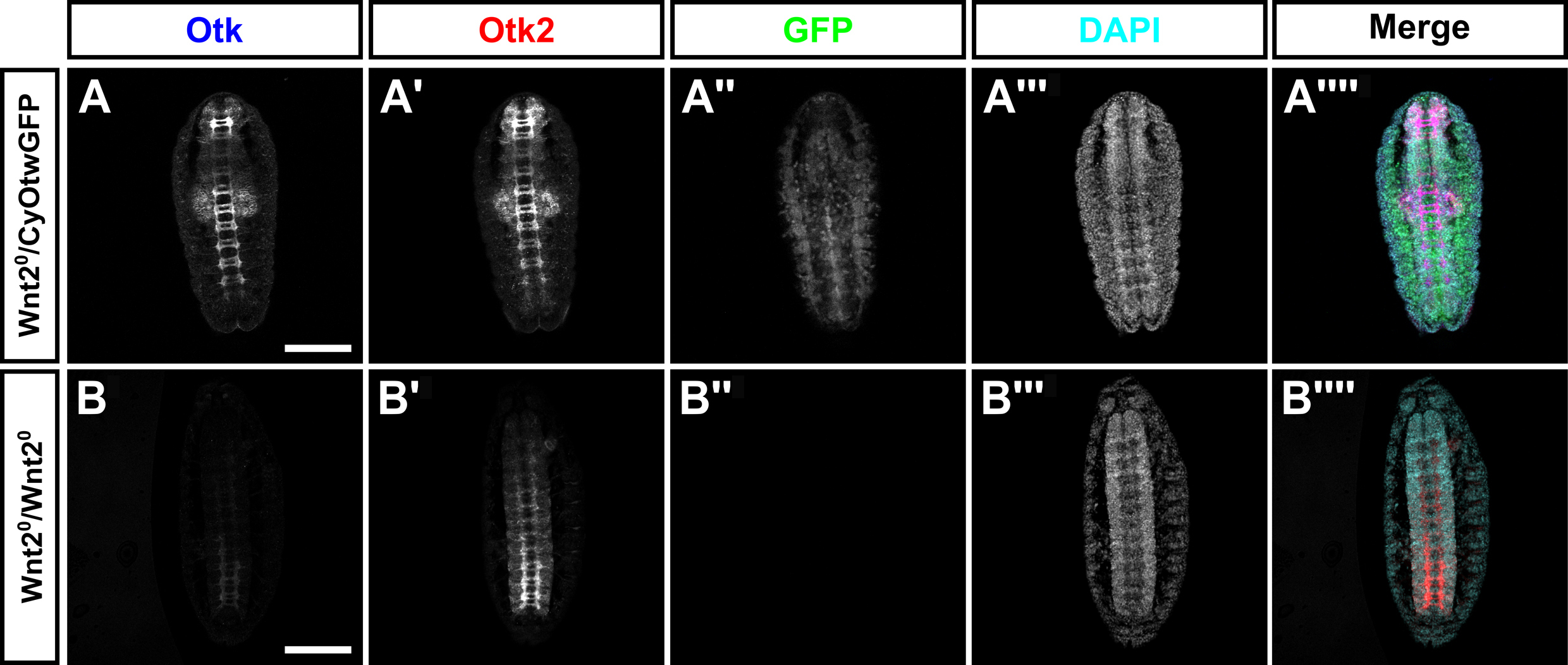

Supplement: Figure S9 — Embryonic expression levels of Otk, but not of Otk2, are dependent on Wnt2. (A–A″″) An embryo heterozygous for Wnt2O and the balancer chromosome Cyo, twist::GFP was stained for Otk (A), Otk2 (A′), GFP (A″) and DAPI (A‴). (B–B″″) A homozygous Wnt2O mutant embryo (marked by the absence of GFP expression from the balancer) stained with the same antibodies as in (A–A″″) was imaged at the same settings as in (A–A″″). Note that levels of Otk are strongly reduced, whereas Otk2 levels are unaffected. Merged images are shown in (A″″, B″″). Scale bar = 50 µm. Anterior is to the top. (JPG) [file pgen.1004443.s009.jpg]

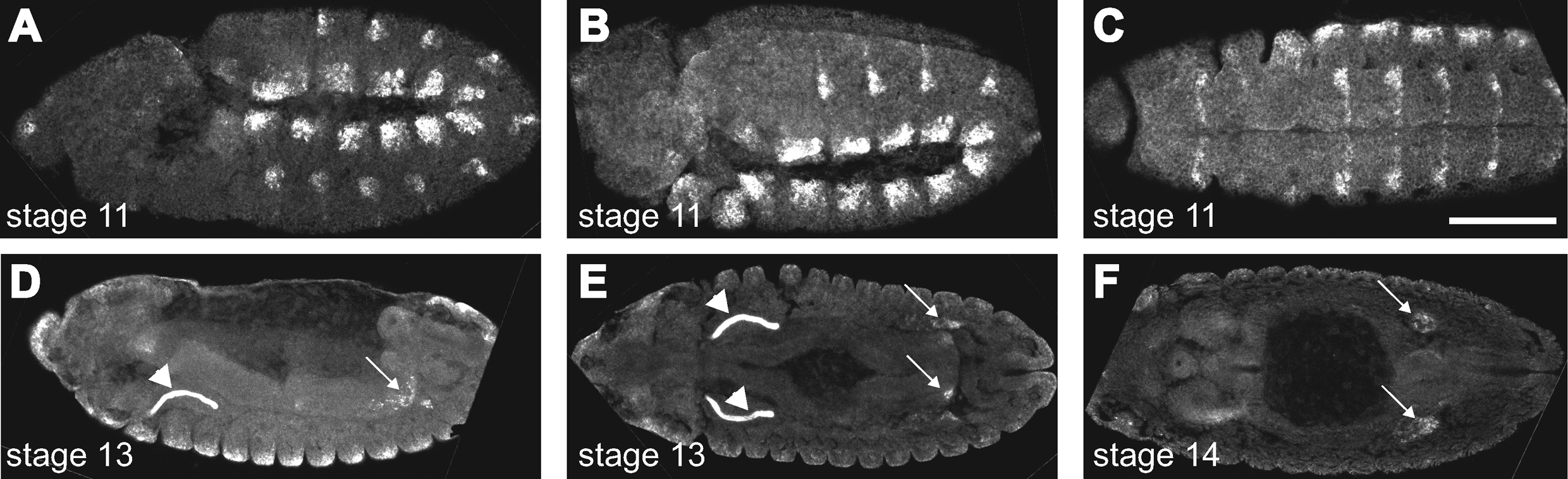

Supplement: Figure S10 — Embryonic expression of Wnt2. Whole mount in situ hybridization reveals the embryonic expression pattern of Wnt2 in a segmentally repeated pattern in the dorsal and ventral epidermis (A–C) and in the developing gonad (arrows in D–F). The signal in the salivary glands (arrowheads in [D, E]) is an artifact. Scale bar = 100 µm. (JPG) [file pgen.1004443.s010.jpg]

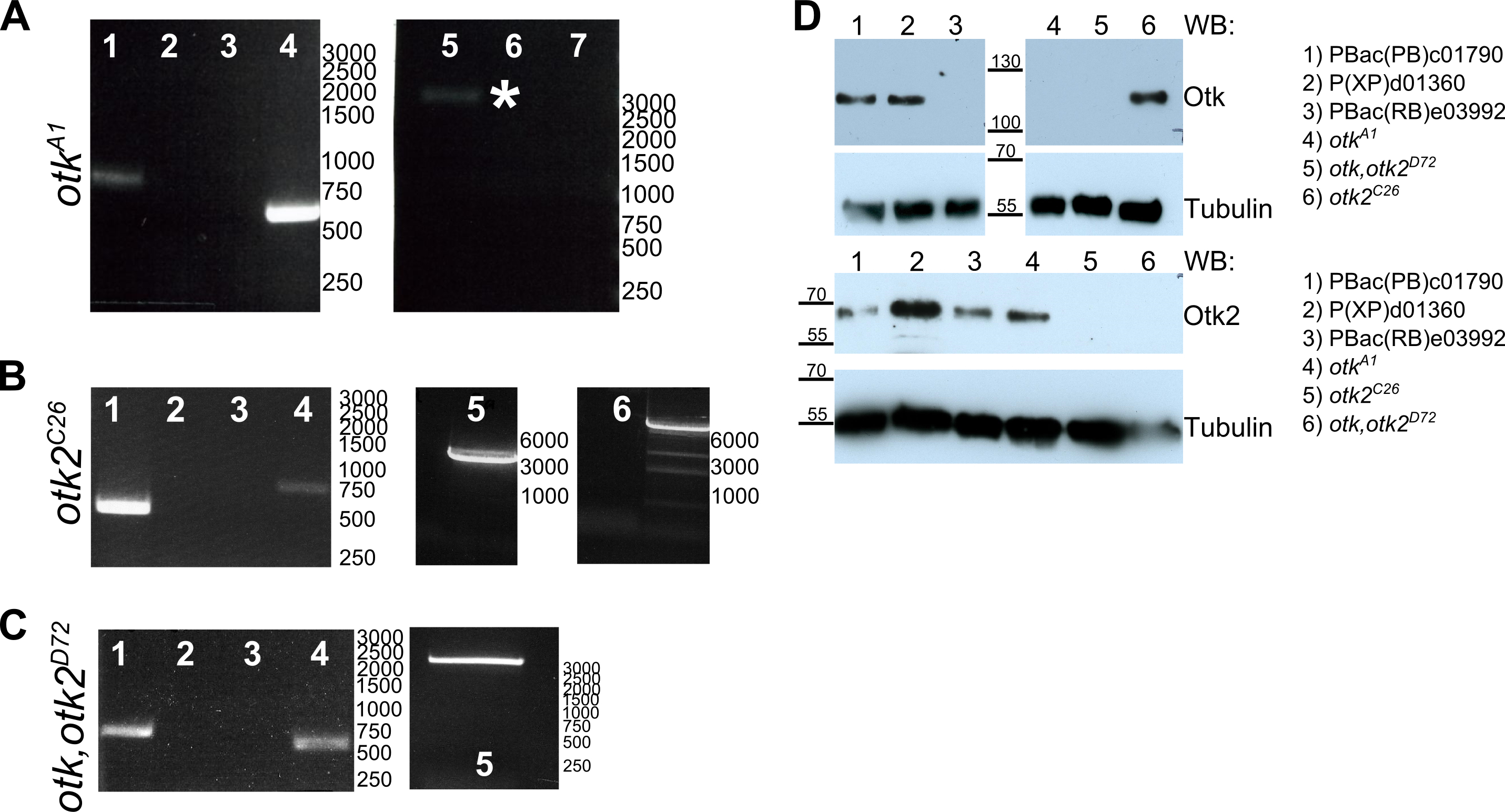

Supplement: Figure S11 — Generation of otk and otk2 null alleles. (A–C) Verification of the three generated alleles by PCR. First, PCRs were done on the transition from the genomic region to the ends of the P-elements to verify that the inner ends of the P-elements are deleted, while the outer ends are still left after recombination has taken place. Furthermore, PCR was performed with primer pairs that encompass the entire deleted regions. All the resulting PCR bands were purified and sequenced. (A) Verification of the otkA1 deletion by PCR on genomic DNA from adult flies. Lane 1–4: The transition between the ends of the P-elements and the genomic region was amplified. PCR was done on the left (1) and right (2) end of P(XP)d01360 and on the left (3) and right (4) end of PBac(RB)e03992. Missing bands indicate the loss of the inner P-element ends. Lane 5 (asterisk): The deleted site was amplified with primers binding upstream and downstream of the whole deletion in the genomic region. The band obtained was purified and sequenced. Adult fly genomic DNA from P(XP)d01360 (lane 6) and PBac(RB)e03992 (lane 7) was used as control. Missing bands can be explained by the large size of the expected fragments. (B) Verification of the otk2C26 deletion by PCR on genomic DNA from adult flies. Lane 1–4: The transition between the ends of the P-elements and the genomic region was amplified. PCR was done on the left (1) and right (2) end of PBac(PB)c01790 and on the left (3) and right (4) end of P(XP)d01360. Missing bands indicate the loss of the inner P-element ends. Furthermore, the deleted site was amplified with primers binding upstream of the deletion in the genomic region and the residual transposon (lane 5) as well as primers binding in the residual transposon and downstream of the deletion in the genomic region (lane 6). The bands obtained were purified and sequenced. (C) Verification of the otk, otk2D72 deletion by PCR on genomic DNA from adult flies. Lane 1–4: The transition between the ends of the [file pgen.1004443.s011.jpg]

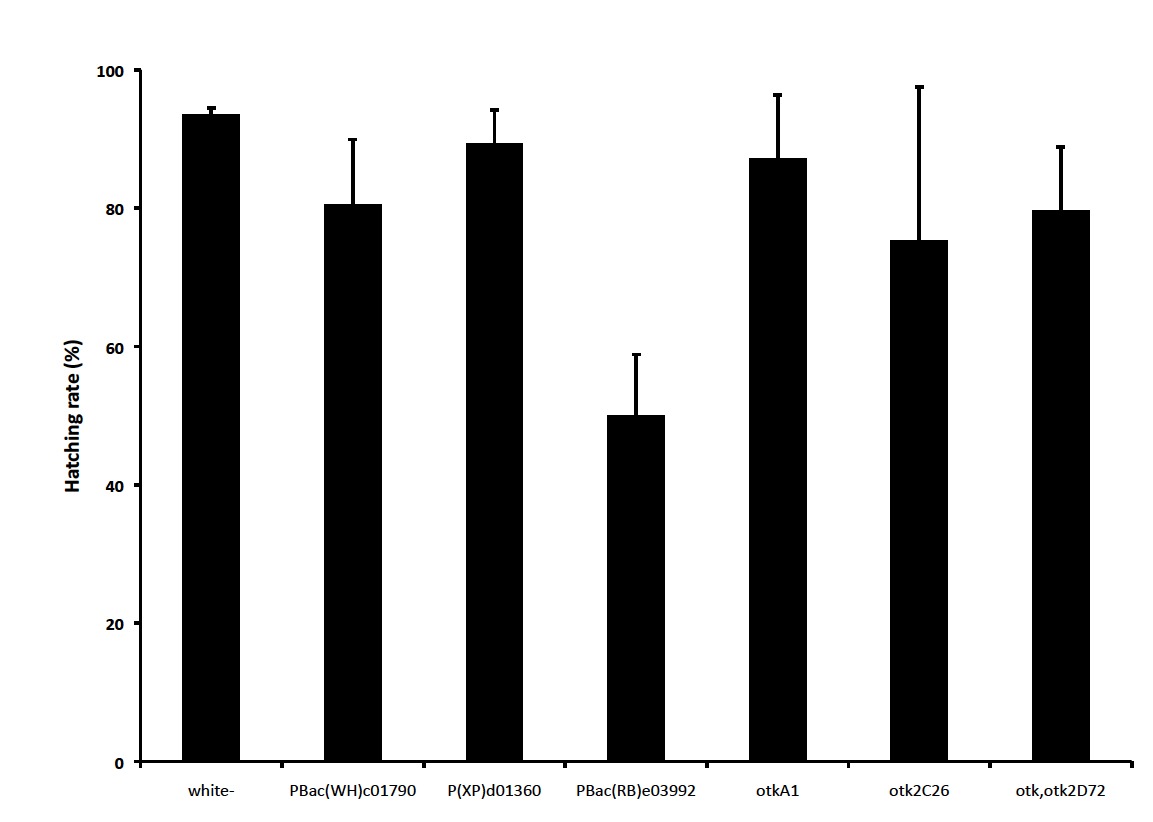

Supplement: Figure S12 — Novel otk and otk2 loss of function mutants are homozygous viable. Lethality assays were performed with embryos of the indicated genotypes. In case of the double deletion, maternally mutant embryos derived from homozygous mutant otk, otk2D72 mothers and heterozygous fathers were used. Embryos were collected and allowed to develop for two days at 25°C. The number of hatched embryos was determined. The experiment was repeated three times. Error bars represent the standard error of the mean. (JPG) [file pgen.1004443.s012.jpg]

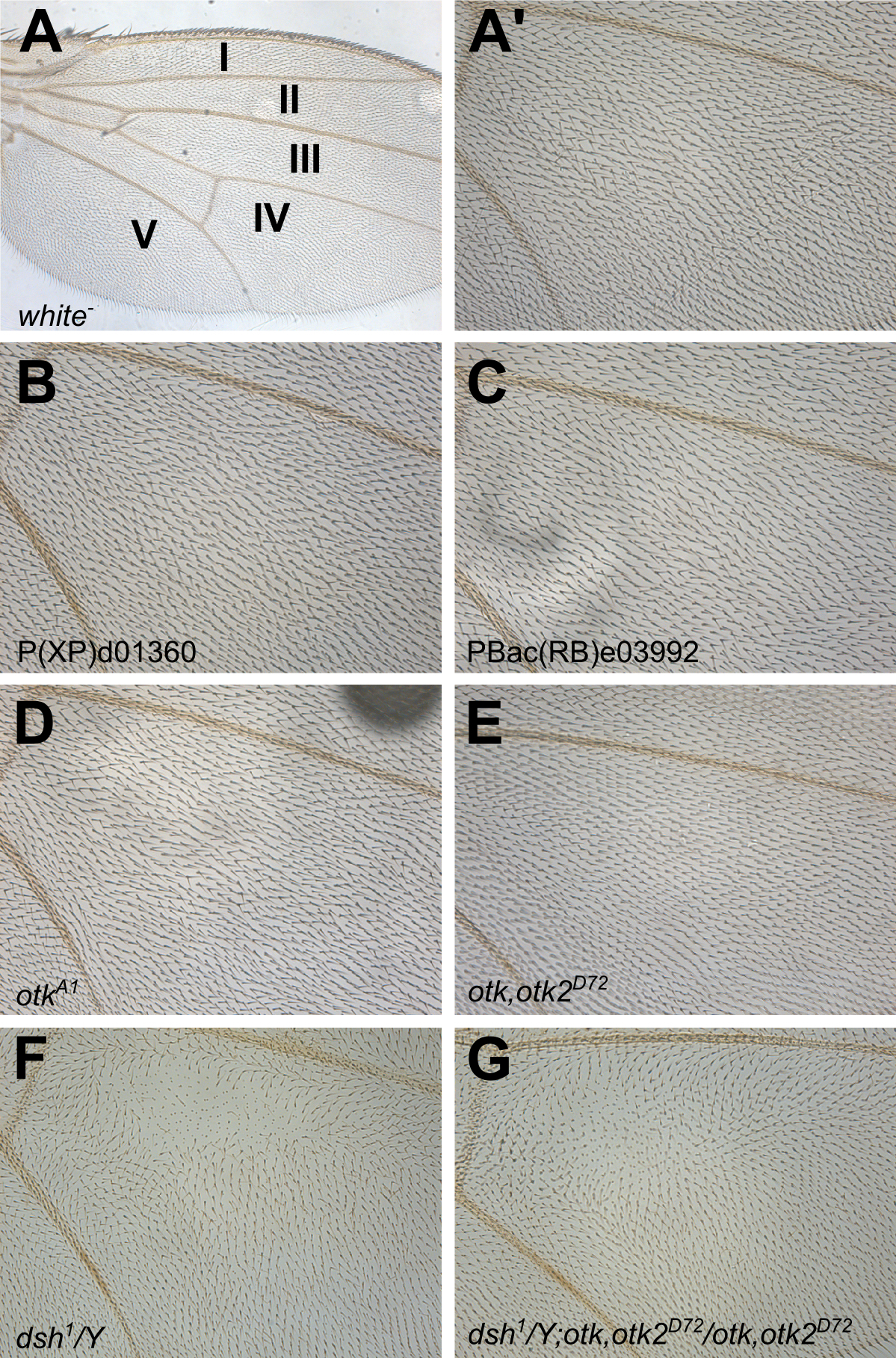

Supplement: Figure S13 — Homozygous otkA1 and otk, otk2D72 flies do not display any wing PCP defects. Defects in planar cell polarity (PCP) signaling usually can be recognized by disorganization of wing hairs as well as thorax and leg bristles of adult flies [59], [60]. In contrast, failure in canonical Wingless signaling leads to wing margin defects [61], [62]. In contrast to the function of PTK7 in vertebrate PCP signaling [12], [18], [19], [23], loss of neither otk nor otk2 alone (C, D; data for otk2 not shown) nor of both otk and otk2 (E) leads to any defects in wing hair orientation compared to w− (A, A′) and the P-element line P(XP)d01360 (B) as controls. (F) Wings from dsh1/Y flies as an example for a characteristic PCP phenotype. The phenotype is unchanged in double mutants for dsh1 and otk, otk2D72 (G). (A) shows a 10× magnification of an adult wing and the five regions of the wing are indicated by roman numerals. (A′, B–G) display 25× magnifications of region IV of the respective wing. Anterior is up and proximal is to the left. (JPG) [file pgen.1004443.s013.jpg]

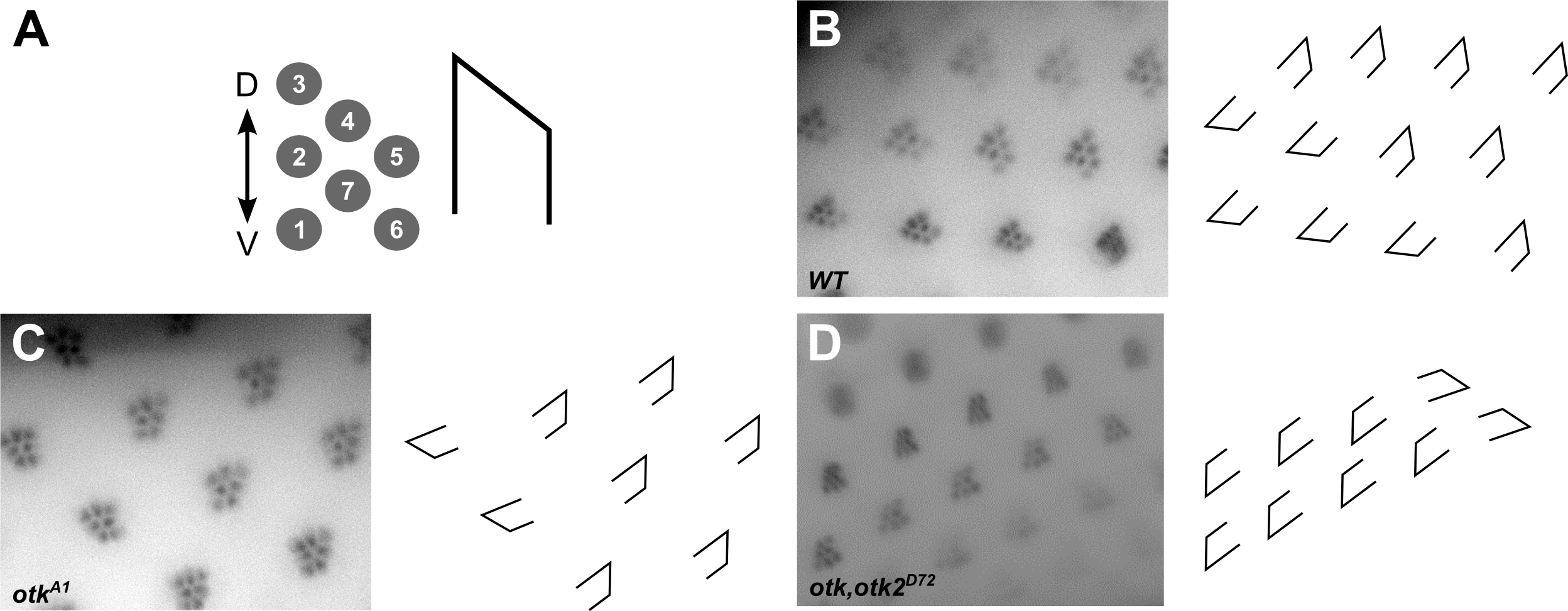

Supplement: Figure S14 — Eyes of homozygous otkA1 and otk, otk2D72 flies do not display any PCP defects. (A) Schematic representation of the ommatidia structure of an adult eye. The photoreceptors in each ommatidium are arranged in an arrow-like manner with the arrow tips pointing away from the equator of the eye (A, B). This organization is frequently disturbed in PCP mutants [59], [60]. Eyes from adult flies homozygous mutant for otkA1 (C) and otk, otk2D72 (D) do not show any defects compared to wild type (B). n = 30 eyes were analyzed for each genotype. 100% of ommatidia showed a wild type orientation in each genotype. (JPG) [file pgen.1004443.s014.jpg]

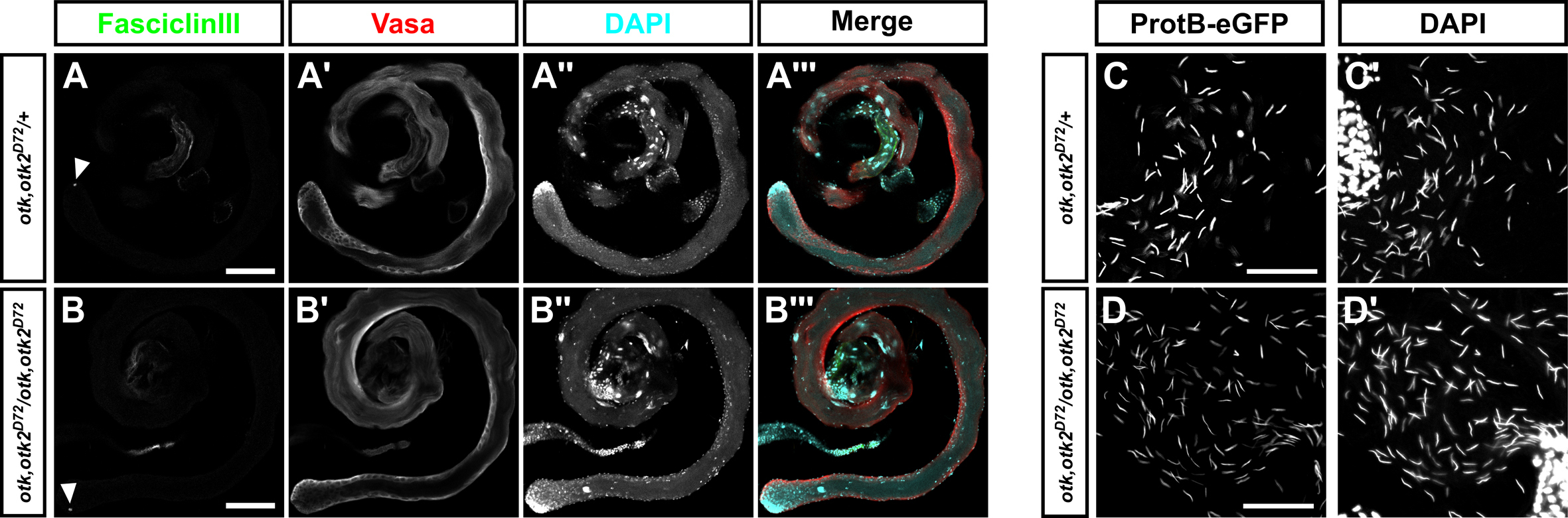

Supplement: Figure S15 — Spermatogenesis in otk, otk2D72 homozygous mutant males. (A–B‴) Adult testes from males heterozygous (A–A‴) and homozygous mutant (B–B‴) for otk, otk2D72 were stained against Fasciclin III (A, B), which marks the hub (arrowhead in A and B) and terminal epithelium, and against Vasa (A′, B′), a marker for germ line stem cells. DAPI staining is shown in (A″, B″), merged images in (A‴, B‴). (C–D′) Higher magnification of sperm in testes from adult males heterozygous (C, C′) and homozygous mutant (D, D′) for otk, otk2D72 carrying a ProtaminB-eGFP transgene. Scale bars: (A, B) = 100 µm, (C, D) = 50 µm. (JPG) [file pgen.1004443.s015.jpg]

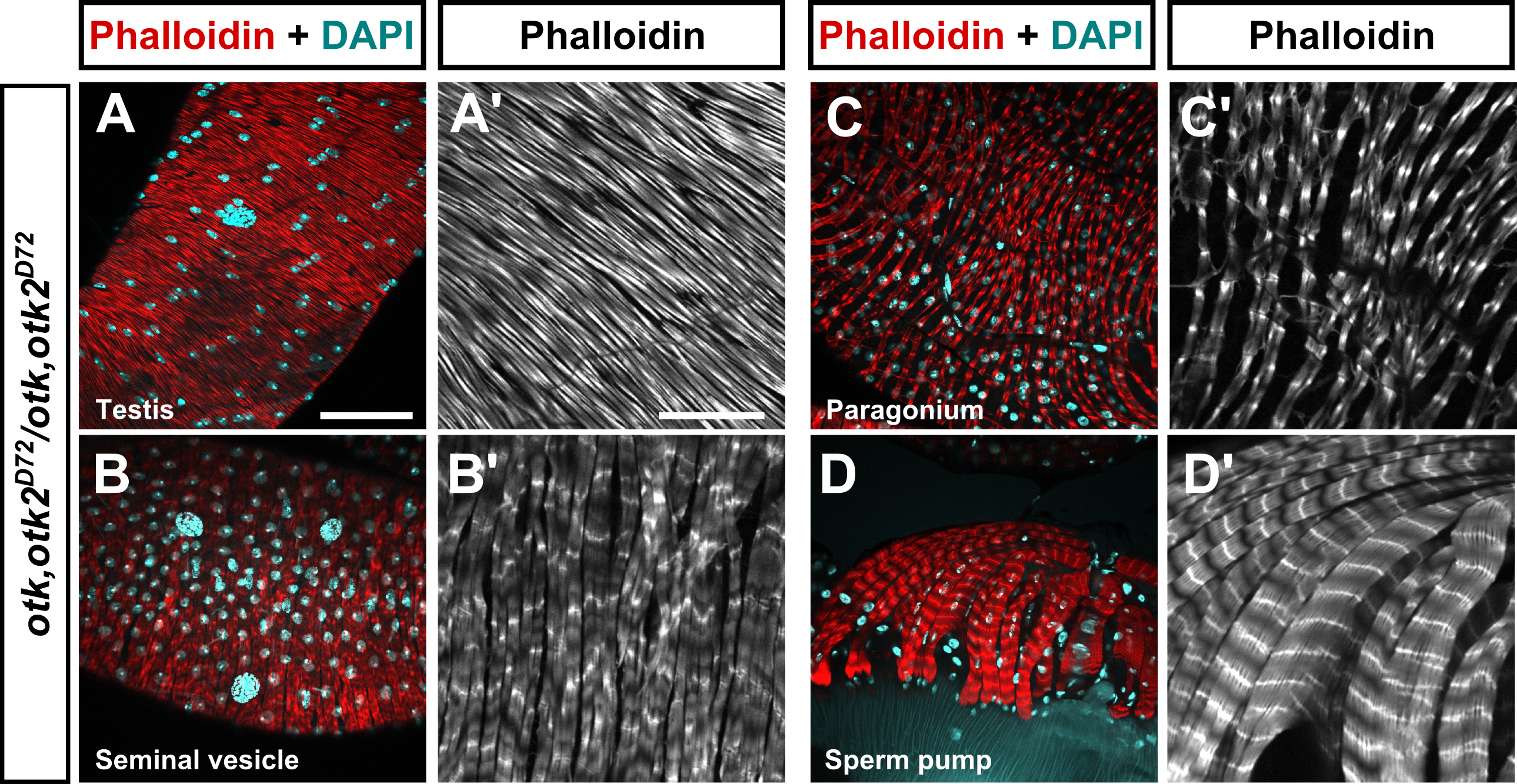

Supplement: Figure S16 — Morphology of the muscle sheath of the male reproductive system of homozygous otk, otk2 mutant animals. The muscle sheaths of the different organs of the male reproductive tract of otk, otk2D72 homozygous mutant males were visualized with Phalloidin for F-actin detection. As described in [46] the testis (A) is surrounded by smooth muscle, whereas the seminal vesicles (B), paragonia (C) and sperm pump (D) are ensheathed by striated musculature. Scale bars: (A–D) = 50 µm; (A′–D′) = 20 µm. (JPG) [file pgen.1004443.s016.jpg]

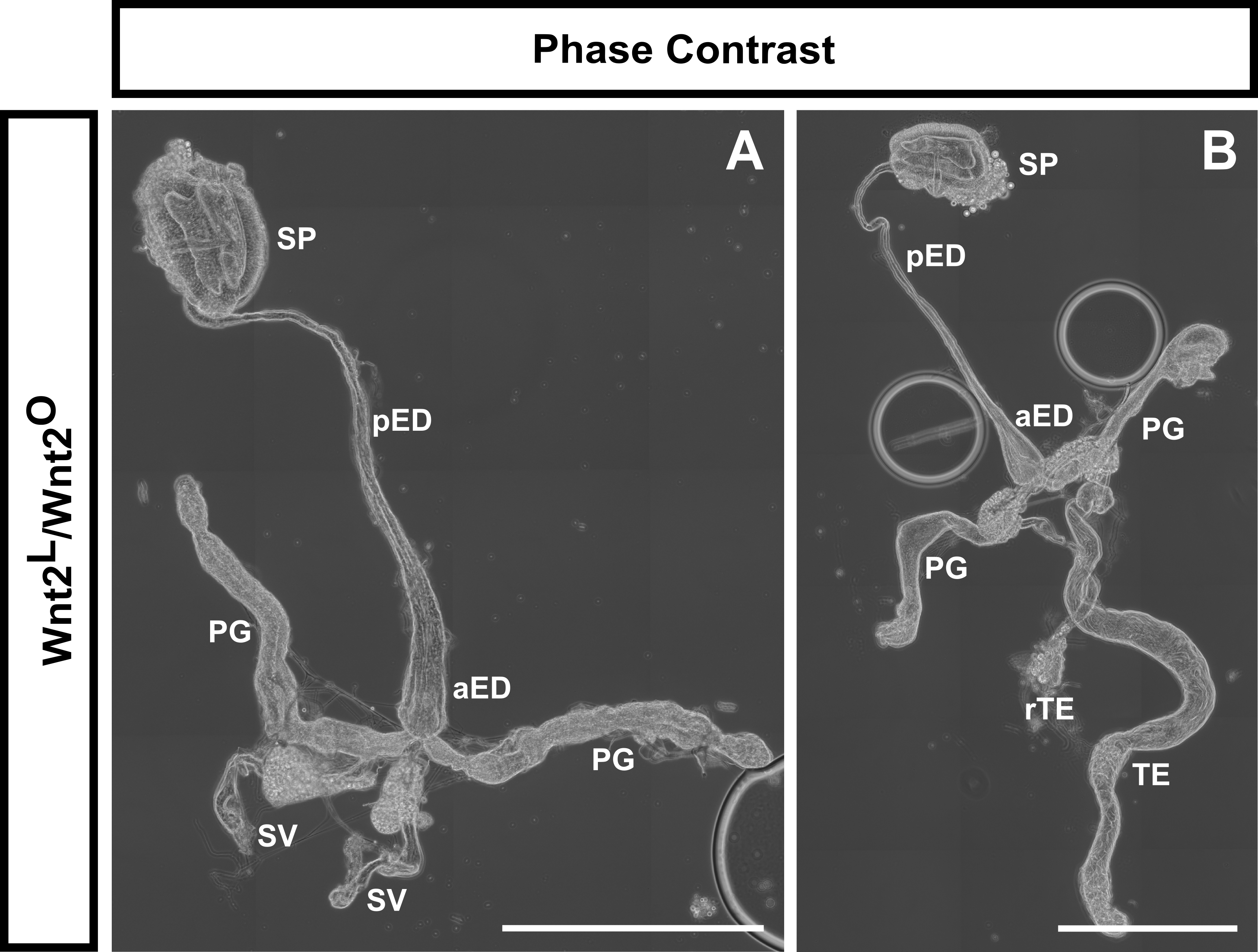

Supplement: Figure S17 — Morphology of the reproductive tract of Wnt2O/Wnt2L transheterozygous males. The Figure shows the variability of the reproductive tract morphology in Wnt2 null mutant males. Whereas the reproductive tract in (A) completely lacks testes, the reproductive tract in (B) has one intact and one rudimentary testis. aED, anterior ejaculatory duct; pED, posterior ejaculatory duct; PG, paragonium (accessory gland); SP, sperm pump; SV, seminal vesicle; TE, testis; rTE, rudimentary testis. Scale bars = 500 µm. (JPG) [file pgen.1004443.s017.jpg]
