# Supplementary material for: The PTK7-Related Transmembrane Proteins Off-track and Off-track 2 Are Co-receptors for Drosophila Wnt2 Required for Male Fertility
Source: PLoS Genet. 2014 Jul 10;10(7):e1004443. doi: 10.1371/journal.pgen.1004443 (PMC4091708; doi:10.1371/journal.pgen.1004443)
Supplement: Table S1 — Fertility of transheterozygous Wnt2 mutant males. (DOCX) [file pgen.1004443.s018.docx]

**Table S1: Fertility of transheterozygous Wnt2 mutant males**

| **Genotype** | **Fertility (%)** | **n =** |
| --- | --- | --- |
| *otk, otk2^D72^/otk, otk2^D72^* | 0 | 9* |
| *Wnt2^L^/Wnt2^0^* | 11 | 36 |
| *Wnt2^I^/Wnt2^0^* | 30 | 10 |
| *Wnt2^I^/Wnt2^L^* | 50 | 4 |
| *Wnt2^L^/Wnt2^RJ^* | 100 | 10 |
| *Wnt2^0^/Wnt2^R^J* | 90 | 10 |
| *Wnt2^RJ^/Wnt2^I^* | 100 | 10 |

* For this genotype many hundred single crosses were evaluated in other experiments that confirmed the complete sterility of these flies.
